# Sex- and age-related differences in LPS-induced lung injury: establishing a mouse intensive care unit

**DOI:** 10.1186/s40635-025-00756-6

**Published:** 2025-05-06

**Authors:** Chantal Crispens, Emilia Fleckenstein, Annett Wilken-Schmitz, Sandra Weber, Michael Gröger, Andrea Hoffmann, Peter Radermacher, Lucy Kathleen Reiss, Steven R. Talbot, Laura Kästner, Kernt Köhler, Kai Zacharowski, Andreas von Knethen, Ulrike Heinicke

**Affiliations:** 1 Department of Anesthesiology, Intensive Care Medicine and Pain Therapy, Goethe University, University Hospital Frankfurt, Theodor-Stern-Kai 7, 60590 Frankfurt am Main, Germany; 2 Institute for Anesthesiological Pathophysiology and Process Engineering, Ulm University, Helmholtzstrasse 8-1, Ulm, Germany; 3 Department of Pharmacology and Toxicology, RWTH Aachen University, 52074 Aachen, Germany; 4 Hannover Medical School, Institute for Laboratory Animal Science, Carl-Neuberg-Straße 1, 30625 Hannover, Germany; 5 Institute of Veterinary Pathology, Justus Liebig University, Frankfurter Str. 96, 35392 Giessen, Germany

**Keywords:** Mechanical ventilation, Critical care, Lipopolysaccharide, Respiratory diseases, Acute lung injury

## Abstract

**Background:**

Mouse models are widely used to establish new therapy concepts for acute lung injury, but the transfer of therapeutic approaches into the intensive care unit often failed. To establish a mouse intensive care unit to adequately reflect the patient’s situation and to investigate sex- and age-related differences in response to lipopolysaccharide.

**Methods:**

For the establishment of a mouse intensive care unit, young (2–3 months) and old (15–18 months) mice of both sexes received continuous respiratory and cardiovascular monitoring for 6 h. Mimicking an acute lung injury by intratracheal lipopolysaccharide stimulation for 6 or 24 h, the impact of sex and age on survival and physiological parameters was evaluated.

**Results:**

The establishment revealed sex- and age-related differences in physiological responses during mechanical ventilation, with old males requiring more noradrenaline to maintain stable hemodynamics. While young mice, irrespective of sex, developed acute lung injury 24 h after lipopolysaccharide administration, old mice exhibited a rapid systemic response, showing signs of lactic acidosis and endotoxemia. Among these, old females had the highest mortality risk, whereas in old males, mechanical ventilation provided effective support, contributing to improved survival outcomes.

**Conclusions:**

We successfully established a mouse intensive care unit that integrated all critical aspects of a human intensive care unit simultaneously. By highlighting sex- and age-related differences following lipopolysaccharide stimulation and mechanical ventilation, our study underscored the need for diversity in preclinical models to improve translation of findings on critical illnesses like acute lung injury into clinical settings.

**Supplementary Information:**

The online version contains supplementary material available at 10.1186/s40635-025-00756-6.

## Background

The intratracheal (i.t.) lipopolysaccharide (LPS) mouse model is widely used and is one of the most established models in preclinical research for studying bacterial pneumonia, acute lung injury (ALI), and acute respiratory distress syndrome (ARDS) [1]. Despite its extensive use, promising therapeutic findings from preclinical studies largely failed to translate into clinical success [2–4]. Preclinical research often focused on survival outcomes without reflecting the complexity of intensive care unit (ICU) management, including organ support and fluid therapy consistent with human guidelines. Additionally, most studies used only young mice of one sex, predominantly male [5, 6]. Historically, sex differences were largely ignored in research, with women underrepresented in clinical trials and experimental studies [7]. While female representation in clinical trials had improved, preclinical models lagged behind, despite their foundational role for clinical research. Additionally, age is an important but often neglected factor in experimental ALI models, despite known age-related differences in ARDS prevalence and outcomes [8, 9]. Furthermore, most preclinical studies used mice that were far too young to accurately reflect the patient population typically treated on ICUs, where ALI/ARDS patients normally received care. [10, 11].

This underrepresentation of female and aged animals weakened the generalizability of findings and reduced the predictive accuracy of preclinical studies [12, 13]. Additionally, omitting sex- and age-stratified analyses hindered understanding of potential biological differences, which may affect therapeutic efficacy, as evidenced in fields such as cardiovascular health, oncology, and mental health [13–16].

Moreover, in preclinical research mice were usually kept in pathogen-free breeding [17], and organ support was minimal or mostly completely absent [2]. Further, fluid management and anesthesia choices diverged from human ICU guidelines, where balanced crystalloids were recommended [18]. These discrepancies highlighted the need for comprehensive preclinical models that more accurately mirror the situation of patients on ICU. Additionally, incorporating sex and age perspectives would improve translational research outcomes and address health inequities.

To address these concerns, we first establish a mouse intensive care unit (MICU) incorporating intravenous (i.v.) anesthesia, balanced crystalloid fluid resuscitation, MV, catecholamine therapy, and continuous monitoring of important physiological parameters, emulating human ICU conditions. The primary objective of this study was to investigate sex- and age-related physiological responses to i.t. LPS administration and MV by assessing disease manifestation, survival, and lung function parameters, including oxygenation, lung compliance, and respiratory mechanics. Hence, to reflect the diversity of the patient collective of a human ICU, young (2–3 months) and old (15–18 months) mice of both sex were included, corresponding to human ages of approximately 19–21 years and 42–78 years, respectively [19].

## Methods

### Study objective

The primary objective of this study was to investigate sex- and age-related physiological responses to i.t. LPS administration and MV by assessing disease manifestation, survival, and lung function parameters, including oxygenation, lung compliance, and respiratory mechanics.

### Mice

Young (2–3 months) and old (15–18 months) C57BL/6 J mice of both sexes (n = 14 per group) were obtained from Janvier-Labs (Le Genest-Saint-Isle, France). Mice were group housed in temperature and humidity controlled cages with a 12:12 h light:dark cycle receiving food and water ad libitum minimum five days before the experiment.

### Anesthesia, surgical instrumentation, and experimental protocol

**Fig. 1:**
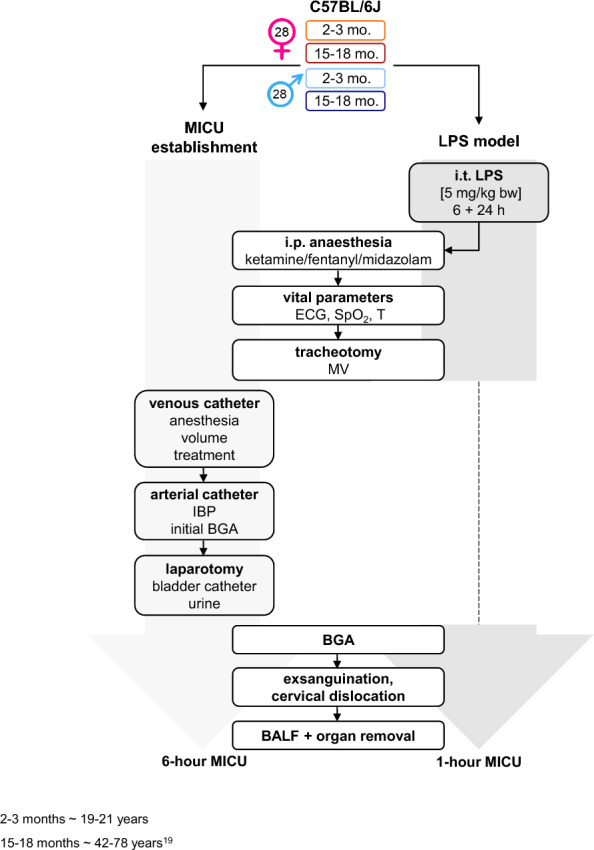
Flow chart. All experiments were carried out according to a chronological order demonstrated by the flow chart. Technical procedures of the MICU establishment (6 h stay) are shown on the left, of the LPS model (1 h stay) on the right, and procedures that occurred in both are depicted in the middle. i.t.: intratracheal; i.p.: intraperitoneal; ECG: electrocardiograph; SpO_2_: oxygen saturation; HR: heart rate; T: temperature; MICU: mouse intensive care unit; h: hour; BGA: blood gas analysis; BALF: bronchoalveolar lavage fluid; MV: mechanical ventilation

The initiation of each animal into the experiment was carried out according to a fixed procedure (Fig. 1). The 6 h MICU corresponded to an ICU stay of approximately 10 days, while the 1 h MICU corresponded to an ICU stay of approximately 1.7 days in a human lifetime [20]. Briefly, mice were anesthetized intraperitoneally (i.p.) with ketamine, midazolam, and fentanyl (ketamine [120 µg/g body weight (bw)], midazolam [1.25 µg/g bw], and fentanyl [0.25 µg/g bw], 10 ml/kg), followed by tracheotomy, and MV using a lung-protective strategy, with continuous monitoring of physiological parameters over 1 h. The complete experimental setup and the comprehensive methodological details were described in the supplement (Supplement Material & Methods, Supplement Fig. 1A–H).

### LPS model

A bacterial lung infection was simulated by i.t. application of 5 µg/g bw LPS (*Escherichia coli* strain (O111:B4); Sigma-Aldrich; #L2630), prepared in ultrapure water, while the animals were unconscious with 3% isoflurane (Piramal Critical Care; #G64E20 A). Afterwards, all mice were scored regularly for weight, visual appearance, mobility, respiration and behavior. If the general condition deteriorated (score ≥ 1) or the endpoint of 6 or 24 h had been reached, the mouse was admitted to the MICU.

### Blood gas analysis (BGA)

For MICU establishment, blood was drawn directly after catheter insertion and at the end of the 6 h MICU. In the LPS model, blood was drawn once at the end of the 1 h MICU. P_a_O_2_, P_a_CO_2_, pH, S_a_O_2_, hemoglobin (Hb), electrolytes (Na^+^, K^+^, Ca^2+^, Cl^−^), glucose (Glu), lactate (Lac) and base excess (BE) were determined with a blood gas analyzer (ViCell MetaFLEX analyzer; Beckman Coulter, Krefeld, Germany).

### Histopathology

The left caudal lobes of the lung were embedded in kerosene wax and sliced into 4 µm sections with a microtome (HistoCore BIOCUT, Leica). The sections were utilized for hematoxylin (H; PanReac AppliChem; 254766.1211) and eosin (E; Roth; X883.1) staining. The images were taken at 100 × magnification with a Leica CRT 5000 microscope. Heart, kidney, liver, and spleen of sham and LPS-stimulated old mice were examined pathologically. The examination was performed quantitatively and in a blinded manner by two independent investigators. Tissue alterations were categorized and calculated as percentage based on the number of mice in the respective group.

### Statistical analysis

Data analysis was performed with the R software v4.0.3 (R Foundation for Statistical Computing, Vienna, Austria) and GraphPad Prism 10.0.2 (GraphPad Software, Boston, Massachusetts, United States). Assuming an α error probability < 0.05, a β error of 20%, an expected difference of 20%, and a standard deviation of 20% within groups, the sample size calculation using G*Power 3.1.3 determined a total of 28 animals (14 per sex and age group) [21].

Kaplan–Meier curves were analyzed using the Log-rank (Mantel-Cox) test with asymmetrical measurement. Continuously collected data were analyzed using a linear mixed effects regression model with sex (two levels: male, female) and age (two levels: young, old) and for MICU establishment also for phase (two levels: initial phase (t = 60 min), experimental phase (t = 60 ≤ 360 min) variables as fixed effects. The mouse IDs were used as random effects to model the within-subjects’ correlations in the data. We also explored the three-way interactions of the variables to assess the between and within-between relationships. The linear model was subsequently transformed into an ANOVA table using Satterthwaite's approximation for the degrees of freedom in the presence of interactions. The ANOVA table identified significant model coefficients in each MICU variable. Post hoc tests were performed with pairwise estimated marginal means for single coefficients and their interactions. In the case of multiple testing, the Tukey method was used for p-value adjustments. Data are shown as means and 95% confidence interval (CI). BGA data were analyzed using normality and longnormality test. For group comparison, One-Way ANOVA followed by Šídák’s correction for multiple comparison was applied to normality distributed data, while the Kruskal–Wallis test with Dunn’s correction for multiple comparison was used for non-normality distributed data. Data are shown as means and 95% CI. P-values were considered significant at three levels: < 0.05 (*), < 0.01 (**), and < 0.001 (***).

## Results

### Physiological responses and survival during MICU establishment and MV

For MICU establishment, 56 mice entered the phase of MV and were included in the analysis. The establishment of the MICU demonstrated significant age- and sex-related differences in physiological responses during MV (Supplement results). Old males exhibited the highest mortality and required greater noradrenaline support to maintain stable hemodynamics (Supplement Figs. 1H, 3E). Respiratory parameters, including SpO₂ and PIP, stabilized during the experimental phase, though females and older mice required higher VT (Supplement Figs. 2 A, B, 3 A). Blood gas analysis (BGA) revealed acidosis linked to high infusion volumes and elevated chloride from the crystalloid solution, contributing to a reduced anion gap and mild base excess changes, likely due to hemodilution (Supplement Table 1).

**Table 1:** Weight loss in sham and LPS-stimulated mice

	Group	Initial [g]	Final [g]	Weight loss [g]	Weight loss [%]
6 h, female	Sham, young	22.6	21.4	1.2	5.3
	LPS, young	21.8	21.2	0.6	2.9
	Sham, old	28.0	27.8	0.3	0.9
	LPS, old	29.1	28.8	0.4	1.3
6 h, male	Sham, young	28.8	27.6	1.2	4.3
	LPS, young	28.8	27.7	1.1	3.8
	Sham, old	35.5	34.4	1.1	3.0
	LPS, old	34.7	34.2	0.5	1.5
24 h, young	Sham, female	22.4	22.1	0.3	1.5
	LPS, female	22.1	19.4	2.6	11.9
	Sham, male	25.8	25.7	0.1	0.6
	LPS, male	25.2	22.2	3.1	12.1

Data are shown as means: Initial = before LPS application, final = 1 h MICU start

LPS: lipopolysaccharide; MICU: mouse intensive care unit; h: hour

### Age- and sex-dependent responses to i.t. LPS-administration

With this MICU setup, we next wanted to determine whether it would be suitable to investigate a LPS model which simulated a bacterial infection of the lung. Therefore, we first assessed potential sex- and age-related differences in the extent of disease manifestation after i.t. LPS application in young and old mice of both sexes. Hence, 56 mice were i.t. stimulated with LPS for 6 and 24 h. Mice were admitted to a simplified 1 h MICU setup either when their general condition deteriorated (score ≥ 1) or after reaching the 6- or 24 h endpoint. Due to technical and surgical issues, nine mice were eliminated from the experiment (male young n = 3, male old n = 3, female young n = 1, female old n = 2).

Since weight loss is an independent and objective indicator of declining health in mice, bw was recorded before LPS or water administration, and before anesthesia (Table 1). Sham-stimulated mice experienced little to no weight loss (Table 1). However, after 6 h, old sham males showed significantly higher weight loss compared to old sham females (1.1 ± 0.6 g *vs* 0.3 ± 0.4 g; p = 0.011). Crucially, all LPS-stimulated mice experienced significant and progressive weight loss. The most pronounced weight loss occurred 24 h after LPS administration, with a 12.1% reduction in young males and 11.9% in young females. After 6 h, weight loss was highest in young males (3.8%), followed by young females (2.9%), old males (1.5%), and old females (1.3%).

**Fig. 2:**
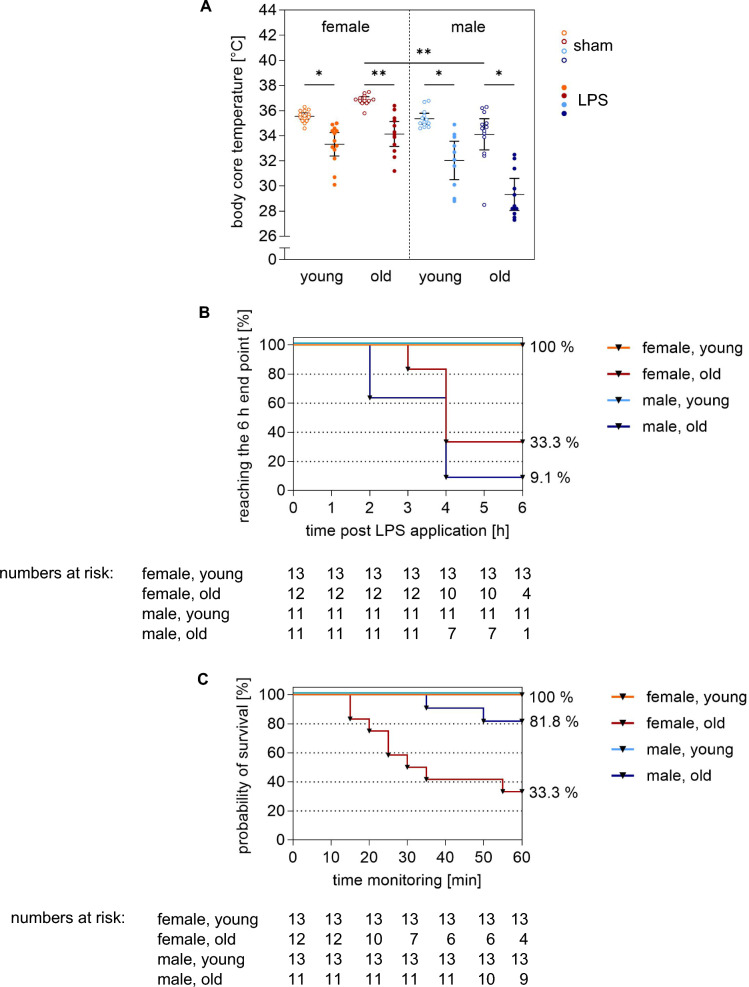
LPS effect on general condition and survival of young and old mice. **A** LPS effect of initial body core temperature in comparison of sex, age and in relation to the corresponding sham groups is shown. **B** Kaplan Meier plot with numbers of risk of young and old LPS-stimulated male and female mice is depicted. Timeslot represented the time *post* application until reaching admission point to the 1 h MICU stay. **C** Kaplan Meier plot with numbers of risk of young and old LPS-stimulated male and female mice is depicted. Timeslot represented the mouse survival during 1 h MICU stay. Data were shown as means ± 95% CI. P-values are calculated with the Kruskal–Wallis test using GraphPad Prism 10.0.2; *p < 0.05, **p < 0.01, ***p < 0.001. Orange: young female mice, red: old female mice, cyan: young male mice blue: old male mice. h: hour; min: minute; LPS: lipopolysaccharide

Accordingly, upon MICU admission, initial body core temperature in all LPS-stimulated mice was significantly lower than in their respective sham groups, with the most substantial decrease observed in old LPS-stimulated males (− 4.8 °C), followed by young males (− 3.3 °C), old females (− 2.7 °C), and young females (− 2.2 °C; Fig. 2A). Interestingly, old sham males had a significantly lower body core temperature at MICU admission compared to old sham females (34.1 (32.9–35.4) °C *vs* 36.9 (36.6–37.1) °C, p = 0.002).

While all young mice of both sexes tolerated LPS administration for both 6 and 24 h, old mice of both sexes exhibited a significantly reduced general condition after LPS stimulation (Fig. 2B). As a result, only four (33.3%) old females and one (9.1%) old male reached the initial 6-h endpoint due to a score of ≥ 1 and were admitted to the MICU earlier (p = 0.105).

These results clearly demonstrated that LPS stimulation in young and old mice of both sexes led to a marked deterioration in general condition, characterized by significant weight loss and reductions in body core temperature. Notably, old mice, especially males, experienced a more severe decline, often requiring premature MICU admission, while young mice tolerated the LPS exposure much better.

### Age- and sex-dependent differences in survival, cardiovascular response, and lung function following LPS stimulation and MV

Given that LPS stimulation led to significant sex- and age-dependent differences in disease manifestation, we next investigated whether these variations affected the response to MV and survival. For this purpose, the results of the MICU establishment were taken into account, and the V_T_ at the beginning of the MV was adjusted individually to the corresponding age and sex (old male: 8.2 µl/g bw; young male: 8.0 µl/g bw; old female: 8.7 µl/g bw; young female: 8.3 µl/g bw) as sex and even small variations in age can influence clinical requirements in respiratory settings (Supplement Fig. 2B). This adjustment helps optimizing ventilatory support while minimizing potential risks associated with over- or under-ventilation. Aiming for a stable EtCO_2_, RR was adjusted individually. Finally, lung injury was assessed by BGA, respiratory mechanics, and lung histology.

All LPS-stimulated young mice of both sexes reached the 6-h endpoint and also survived the subsequent 1 h MICU stay (100%), (Fig. 2B, C). In contrast, the survival rate of LPS-stimulated old mice was lower, with old males showing significantly better survival compared to old females (81.8% *vs* 33.3%; p = 0.01) upon MV. Specifically, 12 (100%) LPS-stimulated old females survived the first 10 min, 10 (83.3%) survived 20 min, 7 (58.3%) survived 30 min, 6 (50%) survived 40–50 min, and 4 (33.3%) survived the full 60 min. Meanwhile, 11 (100%) LPS-stimulated old males survived 40 min, 10 (90.9%) survived 50 min, and 9 (81.8%) survived 60 min, (Fig. 2C).

**Fig. 3:**
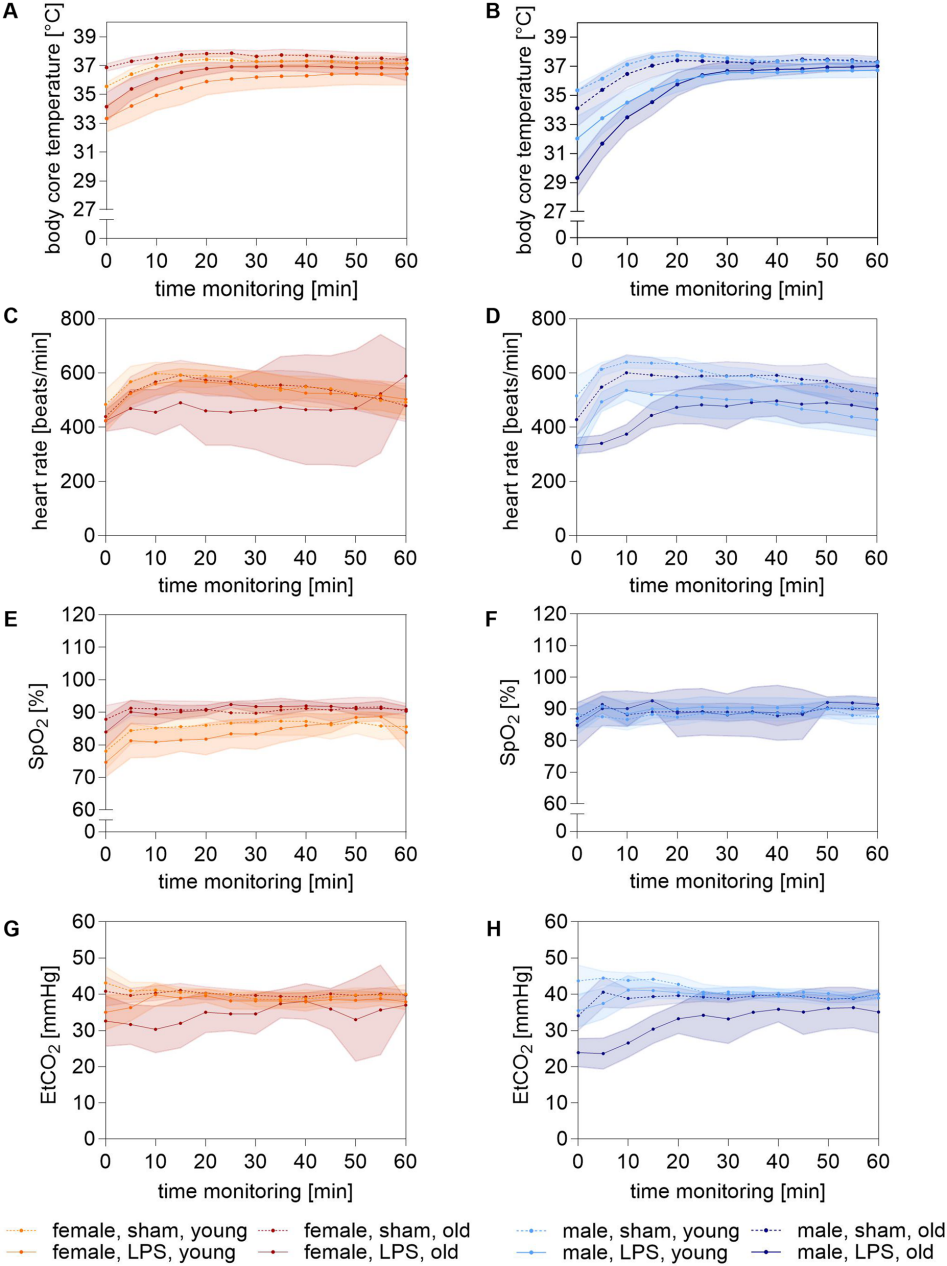
Effects of LPS on ventilation and physiological parameters on 1 h MICU. **A**, **B**, body core temperature [°C], **C**, **D**, HR [beats/min], **E**, **F**, SpO_2_ [%], **G**, **H**, EtCO_2_ [mmHg] shown as progression curves. Data are shown as means ± 95% CI. Orange: young female mice, red: old female mice, cyan: young male mice blue: old male mice. HR: heart rate; min: minute; SpO_2_: oxygen saturation; EtCO_2_: end-tidal carbon dioxide

Next we examined if HR was affected by LPS and whether body core temperature could be stabilized during the 1 h MICU stay. Notably, the low body core temperature observed at the beginning of MV could be stabilized on a normothermic level in all LPS-stimulated mice (Fig. 3A, B). However, during the entire course all sham mice had significantly higher body core temperatures than LPS-stimulated mice. Furthermore, young females tended to maintain lower body core temperatures compared to the corresponding group of old females. In contrast, young males tended to have higher body core temperatures compared to the corresponding group of old males.

Notably, both old and young LPS-stimulated males started with a low HR into the 1 h MICU, stabilizing slower in the old LPS-stimulated males than in the young (Fig. 3D). Old LPS-stimulated females exhibited significantly lower HR compared to old sham females (477 (453–502) beats/min *vs* 536.3 (511–562) beats/min; p = 0.005), (Fig. 3C). However, due to the low survival rates among old LPS-stimulated females, HR values varied considerably throughout the experiment. A similar effect was seen in old LPS-stimulated males compared to old sham males (449 (414–485) beats/min *vs* 564 (535–592) beats/min; p = 0.0004). Young LPS-stimulated males also showed significantly lower HR values compared to young sham males (476 (442–509) beats/min *vs* 582 (555–609) beats/min; p = 0.002).

**Fig. 4:**
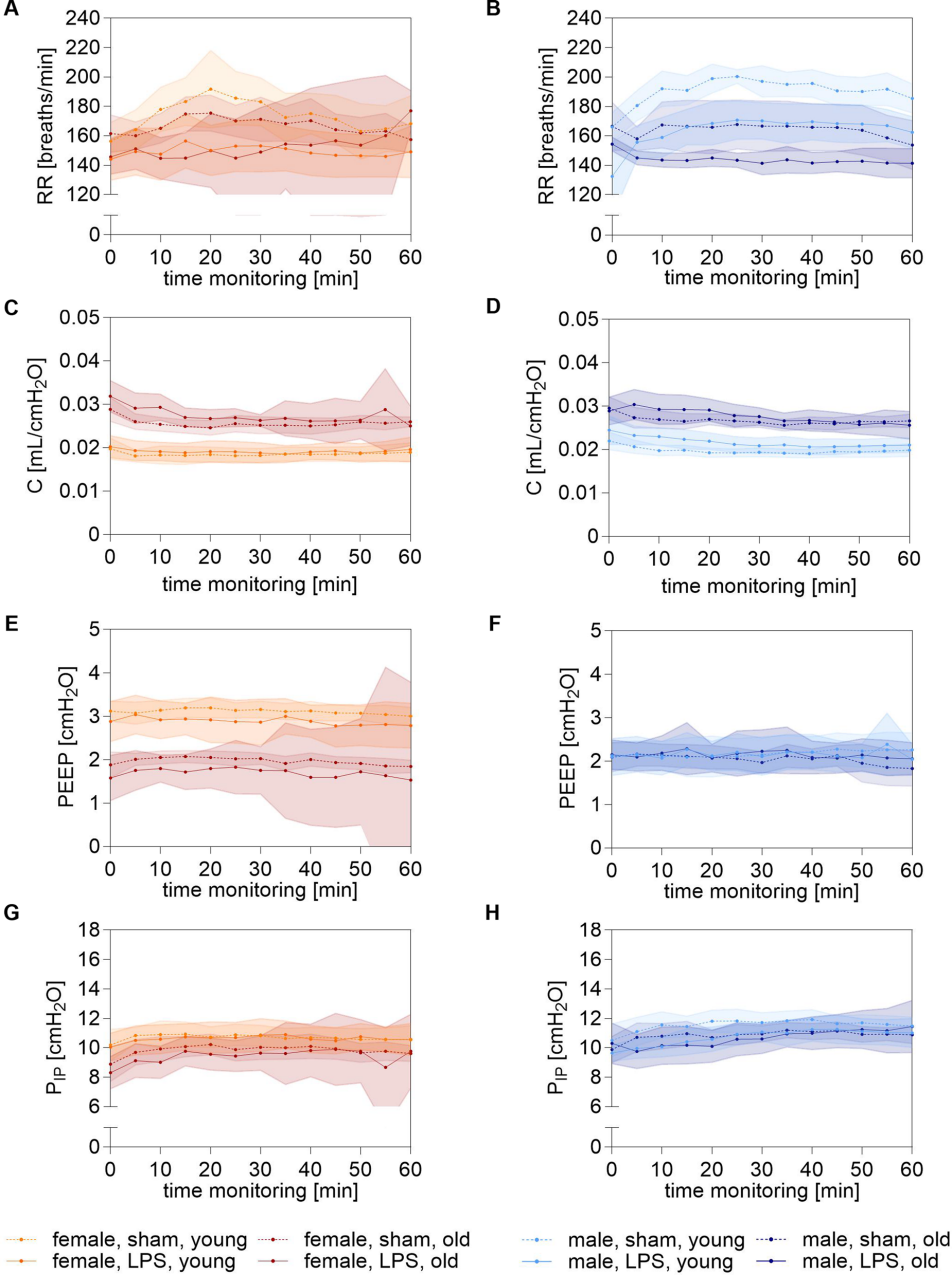
Ventilation parameters on 1 h MICU. **A**, **B**, RR [breaths/min], **C**, **D**, C [ml/cmH_2_O], **E**, **F**, PEEP [cmH_2_O], **G**, **H**, P_IP_ [cmH_2_O] shown as progression curves. Data are shown as means ± 95% CI. Orange: young female mice, red: old female mice, cyan: young male mice blue: old male mice. RR: respiratory rate; C: compliance; PEEP: positive end-expiratory pressure; P_IP_: peak inspiratory pressure; min: minute

Moreover, after an initial drop in SpO_2_ at the beginning of the 1-h MICU stay, levels stabilized across all mice (Fig. 3E, F). However, young females consistently showed lower SpO_2_ values compared to old females, with young sham mice exhibiting significantly lower SpO_2_ compared to old sham females during the 1 h MICU (87 (84–87) % *vs* 91 (90–91) %; p = 0.04). Notably, LPS-stimulated mice started with a lower EtCO_2_ into the 1 h MICU compared to sham mice (p ≤ 0.0001), which was particularly pronounced in old LPS-simulated males (32 (29–35) mmHg *vs* 39 (38–40) mmHg; p = 0.0001), (Fig. 3G, H). Although the EtCO_2_ value could be stabilized in all young LPS-stimulated mice over time, the old LPS-stimulated mice of both sexes remained below the other groups. As a result of the V_T_ adjusted according to sex and age at the beginning of the MV, and the aim of keeping physiological CO_2_ concentration stable, RR was individually adjusted, resulting in fluctuations of RR in all groups (Fig. 4A, B). Thereby, all LPS-stimulated mice had significantly lower RR values compared to their corresponding sham mice (p = 0.0001), which was particularly pronounced in old LPS-stimulated mice (144 (142–146) breaths/min *vs* 164 (161–167) breaths/min; p = 0.0497).

Lung compliance also differed by age (Fig. 4C, D). Old LPS-stimulated females displayed significantly higher compliance compared to young LPS-stimulated females (p ≤ 0.0001), with a similar effect observed between old and young sham females (p ≤ 0.0001). The same pattern was seen in old LPS-stimulated males compared to young LPS-stimulated males (p ≤ 0.0001), and between young and old sham males (p ≤ 0.0001). Despite a stable PEEP and P_IP_ were maintained in all groups during the entire 1-h MICU, PEEP of old females (sham and LPS) were constantly below the PEEP of corresponding young females (sham: p ≤ 0.0001; LPS: p ≤ 0.0001), (Fig. 4E–H). Furthermore, young sham and LPS-stimulated males showed decreased PEEP values compared to the corresponding young female groups (sham: p = 0.015; LPS: p = 0.0004).

These results demonstrated that sex and age significantly influenced survival outcomes, cardiovascular responses, and lung function in LPS-stimulated mice undergoing MV. Notably, upon MV old females had the highest mortality risk, whereas in old males, MV provided effective support, contributing to improved survival outcomes. This difference may indicate a protective effect of MV on the cardiovascular and respiratory stability in young and old LPS-stimulated males, which was not as evident in old LPS-stimulated females.

### Histopathological analysis of mouse organs following LPS-stimulation

**Fig. 5:**
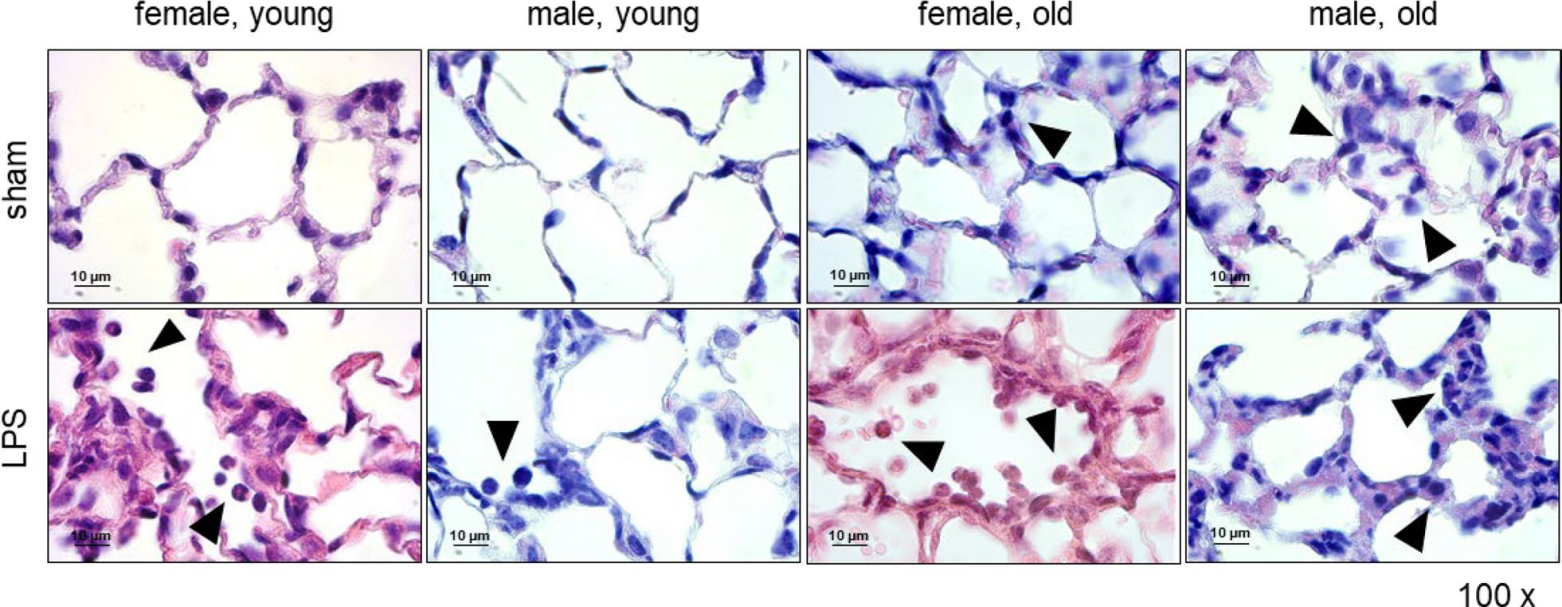
Histopathological analysis of mouse lungs. HE staining of lungs from LPS- and sham- stimulated young and old mice of both sexes is shown. Images were taken at 100 × magnification with Leica CTR 5000 microscope. Arrows indicated infiltrations of immune cells into the alveolar septa and spaces. HE: hematoxylin and eosin; LPS: lipopolysaccharide

HE staining was performed to assess the histopathology of mouse lungs. The staining revealed immune cell infiltration from the bloodstream into the alveolar space in all LPS-stimulated mice (Fig. 5, lower panel). Interestingly, even in old sham males and females, immune cell infiltration was observed in the alveolar space and septa, which was slightly increased in LPS-stimulated old mice of both sexes (Fig. 5). After 24 h (young mice only), a marked accumulation of immune cells in the alveolar septa in both sexes were observed (Supplement Fig. 5A).

Pathological examination of heart, liver, spleen and kidney from old males and females, revealed altered tissue structures in all organs, despite the heart (Tables 2, 3). No significantly altered abnormalities were detected in the organs of old sham compared to old LPS-stimulated mice of both sexes (Table 2). However, immune cell infiltrations were significantly higher in the liver of old LPS-stimulated females (54.6%) compared to old LPS-stimulated males (0%; p = 0.018) (Table 3). Furthermore, a higher fatty degeneration in the liver of LPS-stimulated old females (72.7%) compared to old males (25%; p = 0.07) was tendentially observed, which was significantly increased when comparing female and male sham groups (66.7% *vs* 12.5%; p = 0.05) (Table 3). Additionally, old LPS-stimulated females showed consistently higher hemosiderin storages in the spleen (100%) compared to old males (50%; p = 0.006). In contrast, splenic hematopoiesis was observed more frequently in old LPS-stimulated males (50%) in relation to old females (21.4%; p = 0.204). Additionally, in the kidney of old sham males, single thickened Bowman’s capsules (53.8%) were identified, which was not observed in old sham females (0%; p = 0.005).

**Table 2:** Pathological examination of liver, spleen and kidney from old sham- in comparison of the different stimuli

Organ	Pathology	Male old n (%)		Sham *vs* LPS	Female old n (%)		Sham *vs* LPS
		Sham	LPS	p-value	Sham	LPS	p-value
Liver		n = 8	n = 8		n = 9	n = 11	
	Infiltration of immune cells	2 (25)	0 (0)	0.467	3 (33.3)	6 (54.6)	0.406
	Fatty degeneration	1 (12.5)	2 (25)	1.000	6 (66.7)	8 (72.7)	1.000
	Itocell fatty degeneration	7 (87.5)	8 (100)	0.119	9 (100)	8 (72.7)	0.218
Spleen		n = 10	n = 10		n = 13	n = 14	
	Prominent lymph follicles	10 (100)	10 (100)	1.000	13 (100)	14 (100)	1.000
	Haemosiderin storage	7 (70)	5 (50)	0.650	13 (100)	14 (100)	1.000
	Extramodullary haematopoiesis	6 (60)	5 (50)	1.000	7 (53.8)	3 (21.4)	0.226
Kidney		n = 13	n = 10		n = 12	n = 13	
	Infiltration of immune cells	13 (100)	9 (90)	0.435	12 (100)	13 (100)	1.000
	Single thickened Bowman’s capsules	7 (53.8)	2 (20)	0.197	0 (0)	0 (0)	1.000
	Altered morphology of mesangium	1 (7.7)	2 (20)	0.560	2 (16.7)	1 (7.7)	0.593
	Altered morphology of tubules	11 (84.6)	8 (80)	1.000	9 (75)	12 (92.3)	0.322
	Altered morphology of glomerula	6 (46.2)	4 (40)	0.608	3 (25)	4 (30.8)	1.000

Data are shown as original amount and percentage. P-values are calculated with the Fisher-Exact test

LPS: lipopolysaccharide; n: number; *vs*: versus

^*^p < 0.05

^**^p < 0.01

^***^p < 0.001

**Table 3:** Pathological examination of liver, spleen and kidney from old sham- in comparison of the sexes

Organ	Pathology	Sham male *vs* female n (%)		p-value	LPS male *vs* female n (%)		p-value
		Male	Female		Male	Female	
Liver		n = 8	n = 9		n = 8	n = 11	
	Infiltration of immune cells	2 (25)	3 (33.3)	1.000	0 (0)	6 (54.6)	0.018
	Fatty degeneration	1 (12.5)	6 (66.7)	0.050	2 (25)	8 (72.7)	0.070
	Itocell fatty degeneration	7 (87.5)	9 (100)	0.471	8 (100)	8 (72.7)	0.228
Spleen		n = 10	n = 13		n = 10	n = 14	
	Prominent lymph follicles	10 (100)	13 (100)	1.000	10 (100)	14 (100)	1
	Haemosiderin storage	7 (70)	13 (100)	0.068	5 (50)	14 (100)	0.006
	Extramodullary haematopoiesis	6 (60)	7 (53.8)	1.000	5 (50)	3 (21.4)	0.204
Kidney		n = 13	n = 12		n = 10	n = 13	
	Infiltration of immune cells	13 (100)	12 (100)	1.000	9 (90)	13 (100)	0.435
	Single thickened Bowman’s capsules	7 (53.8)	0 (0)	0.005	2 (20)	0 (0)	0.178
	Altered morphology of mesangium	1 (7.7)	2 (16.7)	0.593	2 (20)	1 (7.7)	0.56
	Altered morphology of tubules	11 (84.6)	9 (75)	0.645	8 (80)	12 (92.3)	0.56
	Altered morphology of glomerula	6 (46.2)	3 (25)	0.411	4 (40)	4 (30.8)	0.685

Data are shown as original amount and percentage. P-values are calculated with the Fisher-Exact test

LPS: lipopolysaccharide; n: number; *vs*: *versus*

^*^p < 0.05, **p < 0.01, ***p < 0.001

These findings demonstrated that, regardless of sex or age, i.t. LPS application activated the immune system and leads to immune cell infiltration from the bloodstream into the alveolar space of the lung. Furthermore, in old females, LPS stimulation resulted in higher fatty degeneration, immune cell infiltration in the liver, as well as increased hemosiderin storage in the spleen, compared to male counterparts.

### Impact of i.t. LPS administration on blood gas parameters in young and old mice of both sexes

**Fig. 6:**
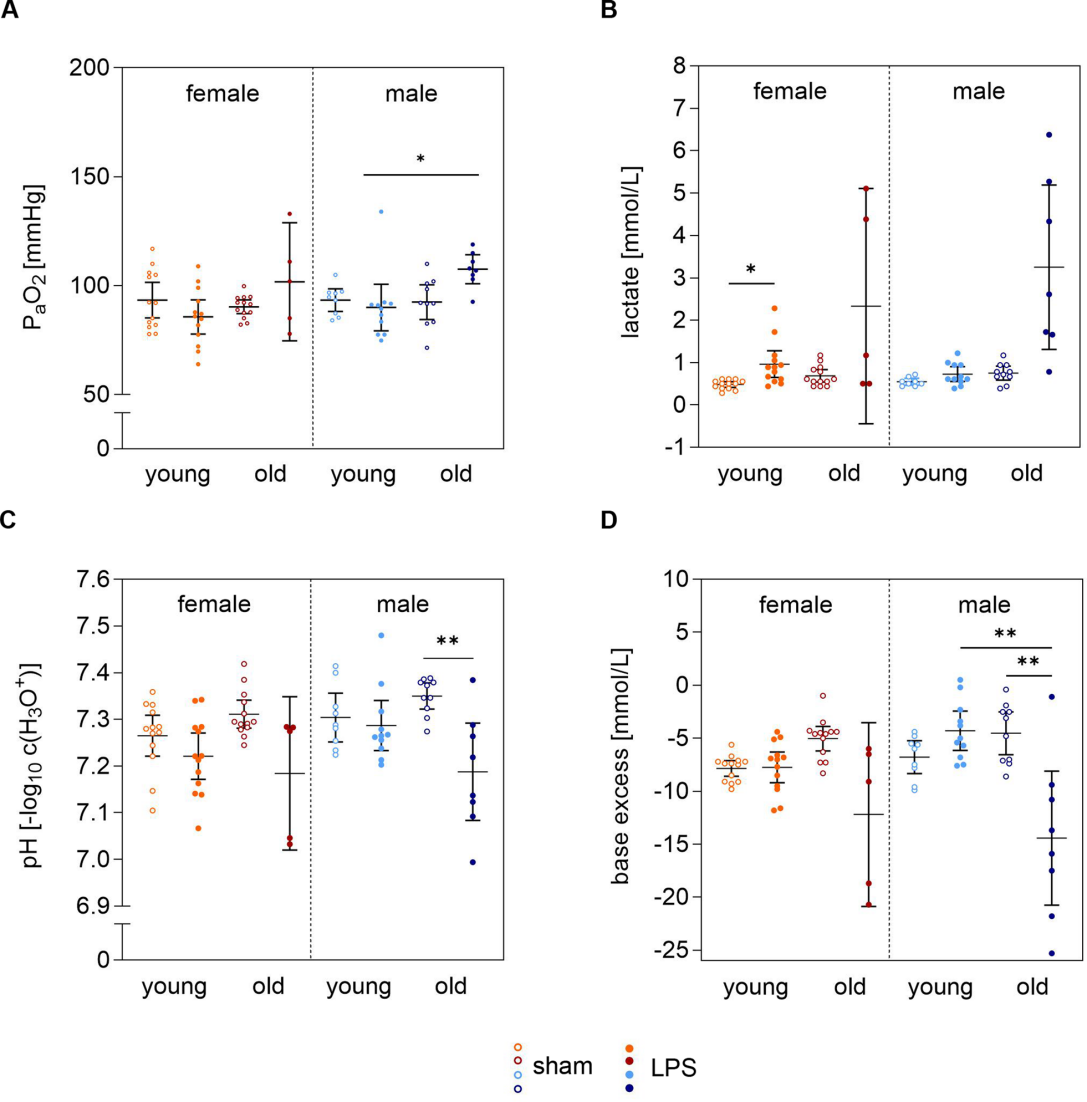
LPS effect on the components of arterial blood after 1 h MICU. BGA results in comparison of sex, age and between LPS and sham stimulated mice. The arterial blood was obtained after the 1 h MICU through an incision of the *carotid artery* and measured immediately with using the ViCell Meta FLEX analyzer, Beckmann Coulter. Data are shown as means ± 95% CI. P-values were calculated with the Kruskal–Wallis test using GraphPad Prism 10.0.2; *p < 0.05, **p < 0.01, ***p < 0.001. **A** P_a_O_2_ [mmHg], **B** lactate [mmol/L], **C** pH [–log_10_ c(H_3_O^+^)], **D** BE [mmol/L]. Orange: young female mice, red: old female mice, cyan: young male mice, blue: old male. MICU: mouse intensive care unit, LPS: lipopolysaccharide; P_a_O_2_: oxygen partial pressure; F_i_O_2_: inspiratory oxygen fraction, BE: base excess

If feasible, BGA samples were obtained at the end of the 1-h MICU to analyze whether LPS influenced gas exchange, pH, and acid–base balance in the blood (Fig. 6A–D, Supplement Table 3, 4). Lung efficiency in oxygen absorption was assessed using P_a_O_2_ (Fig. 6A, Supplement Fig. 5B). Old males exhibited an increase of P_a_O_2_ after 6 h compared to young LPS-stimulated males (108 (101–114) mmHg *vs* 90 (79–101) mmHg; p = 0.014). After 24 h of LPS-stimulation, young females (67 (59–74) mmHg) and young males (69 (61–77) mmHg) showed decreased P_a_O_2_ levels compared to the corresponding sham mice (p = 0.001; p = 0.038).

Additionally, lactate levels rose in all LPS-stimulated mice, with the most pronounced increase in old males (3.3 (1.3–5.2) mmol/L), followed by old females (2.3 (− 0.4–5.1) mmol/L) and young females (1.0 (0.7–1.3) mmol/L; p = 0.025; Fig. 6B). Correspondingly, the pH value decreased in all LPS-stimulated mice, with the most significant drop observed in old males from the corresponding sham mice (7.19 (7.08–7.29); p = 0.006), followed by old females, young females and young males (Fig. 6C). The acidosis observed in old LPS-stimulated mice was associated with decreases in BE, which was significantly most pronounced in LPS-stimulated old males compared to sham mice (−14.4 (−20.8-(−8.1)) mmol/L; p = 0.007) as well as compared to LPS-stimulated young males (−4.3 (−6.1-(−2.4)) mmol/L; p = 0.003). In contrast, BE slightly increased in young LPS-stimulated mice of both sexes (Fig. 6D).

Electrolyte analysis showed significant hyperkaliemia in old, LPS-stimulated males compared to their younger counterparts, while potassium levels were generally lower in LPS-stimulated young females and males. BGA values for all groups are presented in Supplement Table 3 and 4.

The results demonstrated that young mice exhibited impaired lung function, as reflected by a reduced P_a_O_2_ 24 h after LPS-stimulation. In addition, LPS stimulation led to increased lactate levels, particularly in old males, indicating a potential risk of lactic acidosis. The discrepancy between Δlactate and ΔBE can plausibly be explained by the release of other intracellular anions (e.g. phosphate, succinate, malate) resulting from cellular damage and mitochondrial dysfunction. Furthermore, potassium levels were significantly elevated in old, LPS-stimulated males, maybe representing a compensatory mechanism mitigating blood pH disturbances, thereby supporting enhanced survival under inflammatory stress and underlying age- and sex-specific predisposition to hyperkaliemia. These findings underscored significant sex- and age-specific differences in response to LPS stimulation.

## Discussion

In this study we successfully established a MICU with inclusion of mice of both different age and sex, MV, i.v. anesthesia, fluid support, catecholamine treatment, BGA, and continuous monitoring of physiological parameters similarly to the patient’s situation on the ICU. Mimicking an ALI, our LPS model revealed significant differences in disease manifestation based on sex and age. While young mice, irrespective of sex, developed ALI 24 h after LPS administration, old mice exhibited a rapid systemic response showing signs of lactic acidosis suggestive of endotoxemia. Among these, old females had the highest mortality risk, whereas in old males, MV provided effective support, contributing to improved survival outcomes.

Importantly, data from the clinic also showed that males were more susceptible to respiratory infections and had a more severe disease course compared with females [22]. In contrast to our data, mortality was reported to be higher in males compared to females [22–24]. Additional research suggested that men may have a higher incidence of ARDS, but a demographic analysis of the LUNG SAFE study in 2019 found no overall difference in hospital mortality between sexes [25]. However, among severe ARDS cases, women had a higher mortality rate than men [25]. Females with ARDS face unique challenges in management and outcomes, receiving higher VT and being less likely to receive lung-protective ventilation, especially if they are shorter in height [25, 26]. Furthermore, sex-based differences in the treatment of critically ill sepsis patients have been reported, with men being more likely to receive intravenous fluids and oxygen, while women had a 28% higher risk of mortality despite similar adherence to treatment bundles [27]. In addition, the rapid systemic response observed in our model, including signs of lactic acidosis and hypothermia, is consistent with clinical findings where hypothermia has been associated with increased disease severity, higher mortality risk, and poorer outcomes in septic patients [28].

These results are in line with data demonstrating that, similarly to humans, the mortality rate of ALI is enhanced in old compared to young male mice [29, 30]. In addition, a meta-analysis of directly LPS-induced ALI in mammalian species revealed sex-related differences in disease severity, with males exhibiting greater injury than females [13]. In our study, particularly old males experienced a fast and severe decline of deterioration in general condition, with pronounced hypothermia often requiring premature MICU admission. In line with these findings, it had been shown that adult males exhibited more severe sickness behavior and greater body core temperature fluctuations compared to females and pubertal males upon i.p. LPS [10, 11, 13]. Despite the fact that the mice were with nine (adult groups) and five (puberty groups) weeks clearly younger than in our study, these findings highlighted the complex interplay of sex and age in immune responses to LPS in mice.

Sex- and age-specific differences in ventilation outcomes may result in a combination of anatomical, endocrine, and immunological factors [22, 31]. Age-related hormonal changes, such as a decline in estrogen levels in females, could reduce its protective effects on the immune and respiratory systems, while testosterone in males may exert prolonged immunomodulatory benefits. Additionally, immune senescence may accelerate in older mice, particularly females, leading to exaggerated inflammatory responses and increased tissue damage. These factors could contribute to the differential impact of mechanical ventilation in older males.

Lung volumes of female mice are generally higher compared to males, supposedly because of the increased oxygen demand related with possible pregnancy and lactation [32]. Our model used room air and an I:E ratio of 1:1 for MV for all mice, which resulted in lowered SpO_2_ than normal. The used I:E ratio used bore the risk of impaired arterial oxygenation when applying low V_T_ and a PEEP of 2 cmH_2_O [33]. Further, a F_i_O_2_ of 0.21 in mice has been suggested as questionable since mice display a right-shifted oxygen binding curve when compared to humans. Humans can achieve an oxygen saturation of 50% with a P_a_O_2_ of approximately 28 mmHg (P50). In contrast, literature proposed an elevated P50 in mice with 40 mmHg to 71 mmHg [34]. P_a_O_2_ levels around 88 mmHg are presumed normal in awake mice [35]. Another factor of an augmented right shift of the oxygen binding curve is blood acidification during MV and anesthesia [36]. While there were many animal models that promote room air ventilation [37–39], it could be beneficial for the MICU stay if mice, in particular LPS-stimulated old females, who did not benefit from MV, were supplemented with oxygen. Additionally, lung compliance in our model was consistently higher in old mice compared to young mice, irrespective of sex. This finding can be attributed to age-related structural changes, such as the widening of alveolar ducts, which increased air space in the lungs of elderly mice [40].

Interestingly, in old females, LPS stimulation resulted in higher fatty degeneration and immune cell infiltration in the liver, as well as increased hemosiderin storage in the spleen, compared to male counterparts. These findings suggested a heightened inflammatory and metabolic response in old females, potentially indicating sex-specific vulnerabilities to inflammatory stress. Most studies have reported that old mice generally showed increased susceptibility to sepsis, delayed recovery compared to young mice [41, 42], and worse sepsis outcomes in male compared to female animals [43–46]. Similar data were obtained in human studies [47–49]. However, findings are mixed, with some studies indicating worse outcomes in females [27, 50–52] or similar outcomes across sexes [53–55]. Recently, Goswami and Walker demonstrated that old female mice showed a trend towards greater survival of sepsis and a lower disease score compared to old male mice [56]. Intriguingly, closer examination of the mortality curve revealed that old females had faster mortality during the acute phase of sepsis compared to old males [56].

A limitation of our study is that LPS-induced lung injury, while a well-established model for studying inflammation and innate immune responses, does not fully reflect bacterial, viral or aspiration-induced ALI. However, our model provides a valuable tool for investigating immune responses and inflammatory processes central to many forms of ALI/ARDS. Our study also adds to the growing evidence that age and sex significantly influence disease progression and immune response.

Recently, research on sex- and age-related differences had increasingly been incorporated into preclinical mouse studies involving LPS models. However, these studies frequently employed mice that are too young to accurately represent the patient population typically seen in ICUs, and often examined only a single variable—either age or sex—over time. Additionally, critical ICU conditions, such as MV, are seldom replicated in these experimental setups but used as “second hit” to enhance ALI. In our study, we established a MICU that integrated all these critical aspects simultaneously and highlighted the importance of considering both age and sex in preclinical ALI research.

## Conclusions

By highlighting sex- and age-related differences following LPS stimulation, our study underscored the need for diversity in preclinical models to improve translation of findings on critical illnesses like ALI into clinical settings. Our MICU model provides a controlled and reproducible platform to investigate fundamental mechanisms of lung injury, facilitating the study of key pathophysiological mechanisms and biomarker profiles, thereby contributing to the refinement of translational research in critical care medicine. Building on this foundation, targeted, sex- and age-specific therapeutic strategies can now be developed and subsequently evaluated within the complete MICU setup to assess their efficacy and refine patient-specific treatment protocols.

## Supplementary Information

Supplementary Material 1. Figure 1. Overview of the surgical procedures, monitoring, and the survival of the MICU establishment. Intubation was achieved via tracheotomy. Subsequent venousand arterial catheterizationswere performed unilaterally. The bladder was accessed and punctured via laparotomy. Finally, all surgical procedures and connection to monitoring were completed. Surgical procedures C, D, E, and F were only performed for MICU establishment. Survival curve of young and old mice are visualized as Kaplan Meier plot with numbers of risk. The initial phase started with the establishment of MV until the completion of all surgical proceduresand was followed by the experimental phase. Data were collected with a 10 min interval in the time of t = 0 until t = 360. Orange: young female mice, red: old female mice, cyan: young male mice blue: old male mice. MICU, mouse intensive care unit; min, minute. Figure 2. Ventilation parameters on 6-hour MICU. Data of the progression curves were recorded every 10 minutes during the 6-hour MICU by the LabChart 9 software. Data are shown as means ± 95% CI using GraphPad Prism 10.0.2. The initial phase starts with the establishment of MV until the completion of all surgical proceduresand is followed by the experimental phase. A: P_IP_ [cmH_2_O], B: VT [ml/kg], C: RR [breaths/min], D: PEEP [cmH2O], E: C [ml/cmH_2_O]. Orange: young female mice, red: old female mice, cyan: young male mice, blue: old male mice. P_IP_, peak inspiratory pressure; PEEP, positive end-expiratory pressure; VT, tidal volume; RR, respiratory rate; C, compliance; min, minute. Figure 3. Ventilation and physiological parameters on 6-hour MICU. SpO_2_, EtCO_2_, HR, MAP, NA, body core temperatureshown as progression curves. The initial phase started with the establishment of MV until the completion of all surgical proceduresand was followed by the experimental phase. Data were collected with a 10 min interval in the time of t = 0 until t = 360. Values are depicted as means and 95% CI. Orange: young female mice, red: old female mice, cyan: young male mice blue: old male mice. EtCO_2_, end-tidal carbon dioxide; HR, heart rate; min, minute; MAP, mean arterial pressure; NA, noradrenaline; SpO_2_, oxygen saturation. Figure 4. Creatinine clearance after 6 h of MICU. To calculate Cl_Crea_, urine was collected, and creatinine levels were measured in urine and plasma, 1, 3, 8). Data are shown as means and 95% CI. Statistical differences were assessed using the Kruskal-Wallis test using GraphPad Prsim 10.0.2; *p ˂ 0.05, **p ˂ 0.01, ***p ˂ 0.001. Figure 5. LPS-stimulated immune cell migration and PaO_2_ of young mice 24 h after LPS application. LPS and sham stimulated young female and male mice in pathological comparison of their lung morphologies, as well as their PaO_2_ levels. A: HE stained mouse lungs. Images were taken at 100x magnification with Leica CTR 5000 microscope. Arrows indicated infiltrations of immune cells into the alveolar septa. B: PaO_2_ [mmHg]. Data of the PaO_2_ was calculated using final BGA resultsafter the 1-hour MICU using the ViCell Meta FLEX analyzer, Beckmann Coulter. Data were shown as means and 95% CI. Statistical differences were assessed using the Kruskal-Wallis test using GraphPad Prsim 10.0.2; *p ˂ 0.05, **p ˂ 0.01, ***p ˂ 0.001

Supplementary Material 2

## Data Availability

The datasets used and/or analyzed during the current study are available from the corresponding author upon reasonable request.

## Abbreviations

µg: Microgram
µm: Micrometer
ALI: Acute lung injury
ARDS: Acute respiratory distress syndrome
BALF: Broncho alveolar lavage fluid
BE: Base excess
BGA: Blood gas analysis
BI: Bacterial infection
BSA: Bovine serum albumin
bw: Body weight
C: Compliance
Ca^2+^: Calcium
CI: Confidence interval
Cl^−^: Chloride
Cl_Crea_: Creatinine clearance
cm: Centimeter
dL: Deciliter
ECG: Electro-cardiogram
EtCO_2_: End-tidal carbon dioxide
F_i_O_2_: Fraction of inspired oxygen
g: Gram
Glu: Glucose
h: Hour
H_2_O: Water, water
Hb: Hemoglobin
HCO_3_^−^: Bicarbonate
HE: Hematoxylin and eosin
HR: Heart rate
i.e.: *id est*
i.p.: Intraperitoneal
i.t.: Intratracheal
i.v.: Intravenous
I:E: Inspiratory to expiratory
ICU: Intensive care unit
K^+^: Potassium
kg: Kilogram
L: Liter
Lac: Lactate
LPS: Lipopolysaccharide
MAP: Mean arterial pressure
MICU: Mouse intensive care unite
min: Minute
mL: Milliliter
mmHg: Millimeter of mercury
mmol: Millimole
MV: Mechanical ventilation
n: Number
NA: Noradrenaline
Na^+^: Sodium
P_a_CO_2_: Carbon dioxide partial pressure
P_a_O_2_: Oxygen partial pressure
PBS: Phosphate buffered saline
PEEP: Positive end-expiratory pressure
PFA: Formaldehyde
pH: Pondus hydrogenii
P_IP_: Peak inspiratory pressure
RM: Recruitment maneuvers
RT: Room temperature
S_a_O_2_: Arterial oxygen saturation
SpO_2_: Oxygen partial pressure
t: Time
T: Temperature
VCV: Volume-controlled ventilation
*vs*: Versus
V_T_: Tidal volume
