# Supplementary material for: Sex- and age-related differences in LPS-induced lung injury: establishing a mouse intensive care unit
Source: Intensive Care Med Exp. 2025 May 6;13:48. doi: 10.1186/s40635-025-00756-6 (PMC12055714; doi:10.1186/s40635-025-00756-6)
Supplement: Supplementary file 1 — Supplementary Material 1. Figure 1. Overview of the surgical procedures, monitoring, and the survival of the MICU establishment. Intubation was achieved via tracheotomy. Subsequent venousand arterial catheterizationswere performed unilaterally. The bladder was accessed and punctured via laparotomy. Finally, all surgical procedures and connection to monitoring were completed. Surgical procedures C, D, E, and F were only performed for MICU establishment. Survival curve of young and old mice are visualized as Kaplan Meier plot with numbers of risk. The initial phase started with the establishment of MV until the completion of all surgical proceduresand was followed by the experimental phase. Data were collected with a 10 min interval in the time of t = 0 until t = 360. Orange: young female mice, red: old female mice, cyan: young male mice blue: old male mice. MICU, mouse intensive care unit; min, minute. Figure 2. Ventilation parameters on 6-hour MICU. Data of the progression curves were recorded every 10 minutes during the 6-hour MICU by the LabChart 9 software. Data are shown as means ± 95% CI using GraphPad Prism 10.0.2. The initial phase starts with the establishment of MV until the completion of all surgical proceduresand is followed by the experimental phase. A: PIP [cmH2O], B: VT [ml/kg], C: RR [breaths/min], D: PEEP [cmH2O], E: C [ml/cmH2O]. Orange: young female mice, red: old female mice, cyan: young male mice, blue: old male mice. PIP, peak inspiratory pressure; PEEP, positive end-expiratory pressure; VT, tidal volume; RR, respiratory rate; C, compliance; min, minute. Figure 3. Ventilation and physiological parameters on 6-hour MICU. SpO2, EtCO2, HR, MAP, NA, body core temperatureshown as progression curves. The initial phase started with the establishment of MV until the completion of all surgical proceduresand was followed by the experimental phase. Data were collected with a 10 min interval in the time of t = 0 until t = 360. Values are depicted as means a [file 40635_2025_756_MOESM1_ESM.pptx]

## Slide 1
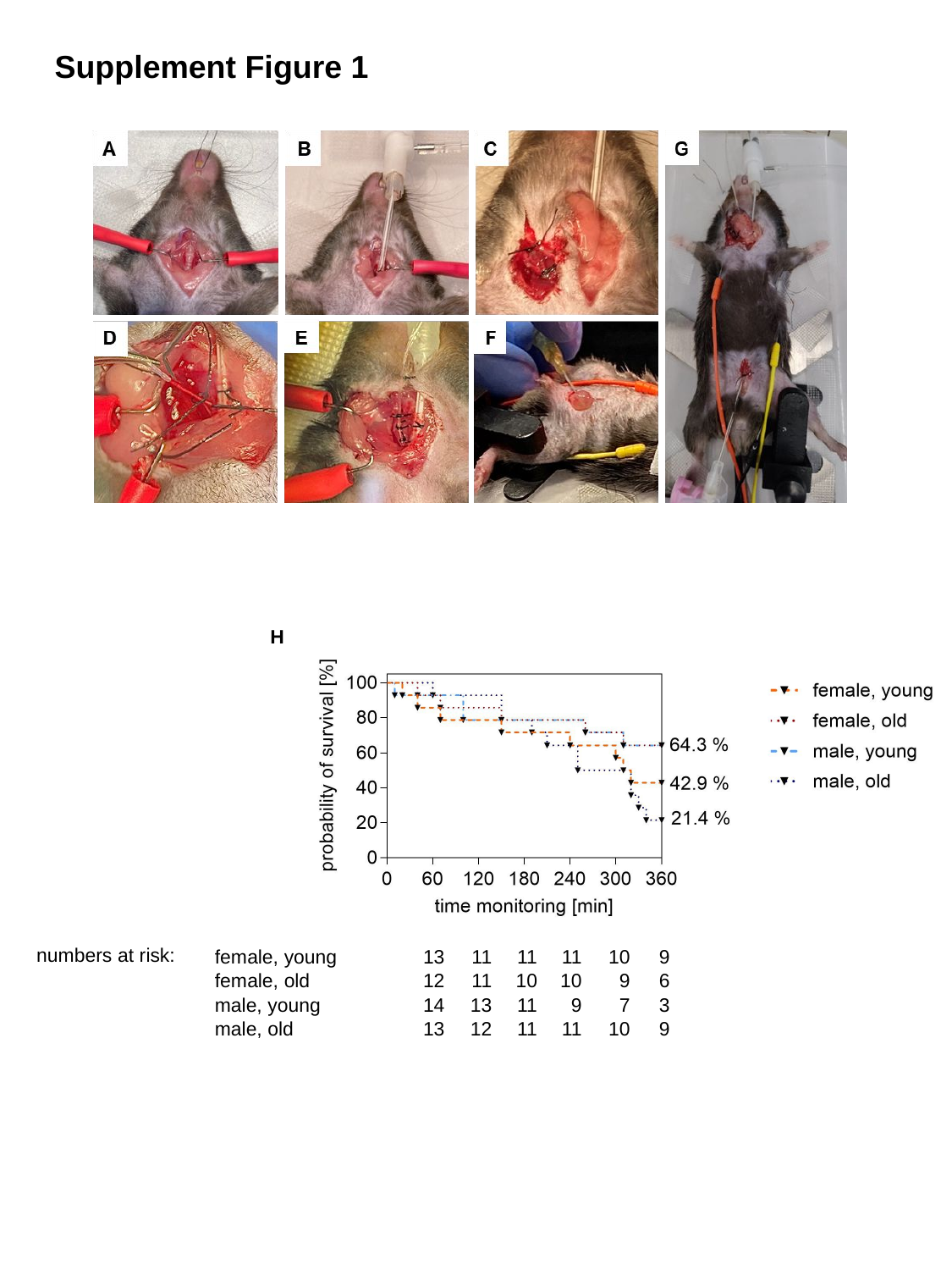

Supplement Figure 1
H
numbers at risk:
| female, young | 13 | 11 | 11 | 11 | 10 | 9 |
| --- | --- | --- | --- | --- | --- | --- |
| female, old | 12 | 11 | 10 | 10 | 9 | 6 |
| male, young | 14 | 13 | 11 | 9 | 7 | 3 |
| male, old | 13 | 12 | 11 | 11 | 10 | 9 |

## Slide 2
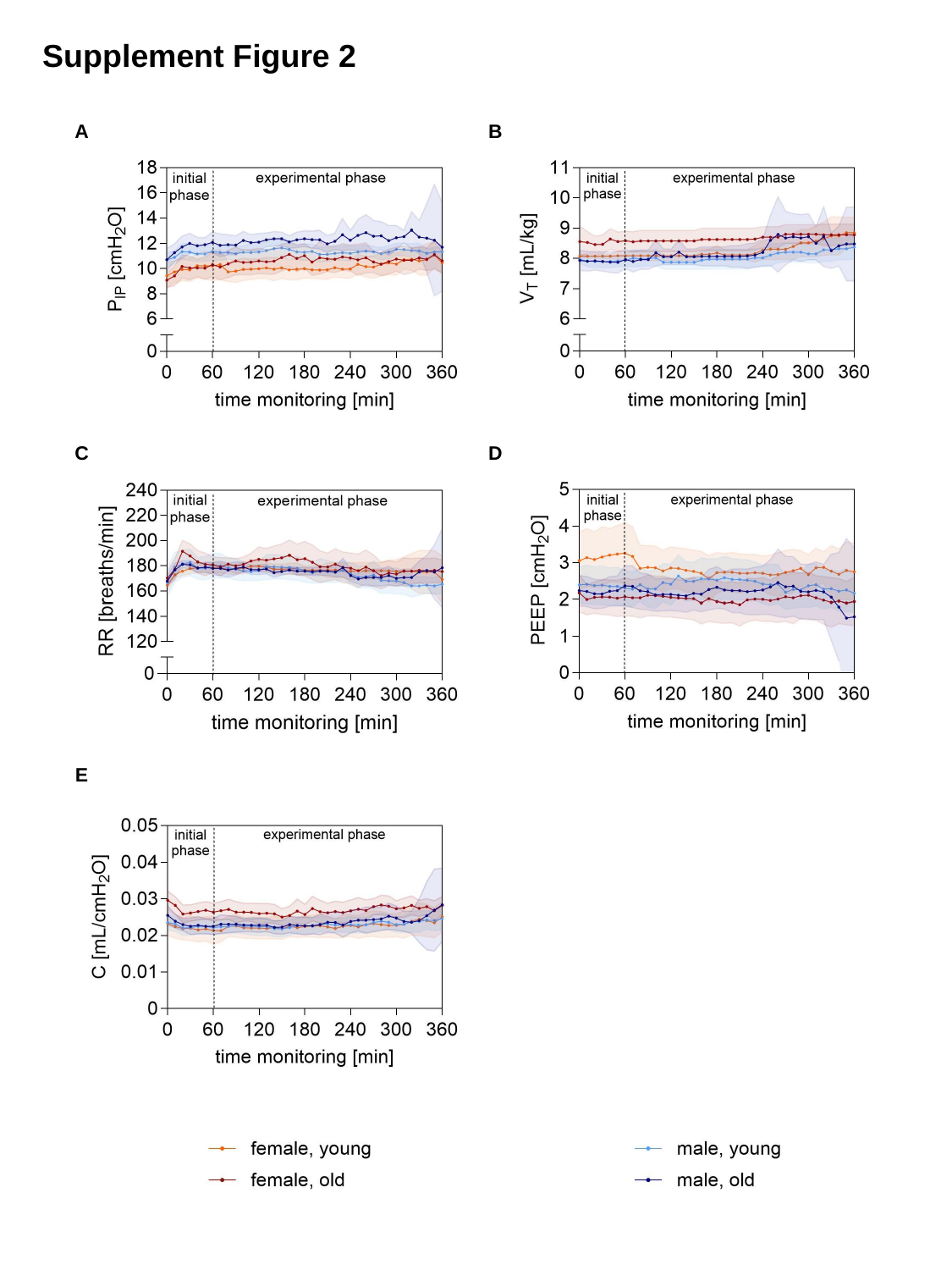

Supplement Figure 2
A
B
C
D
E

## Slide 3
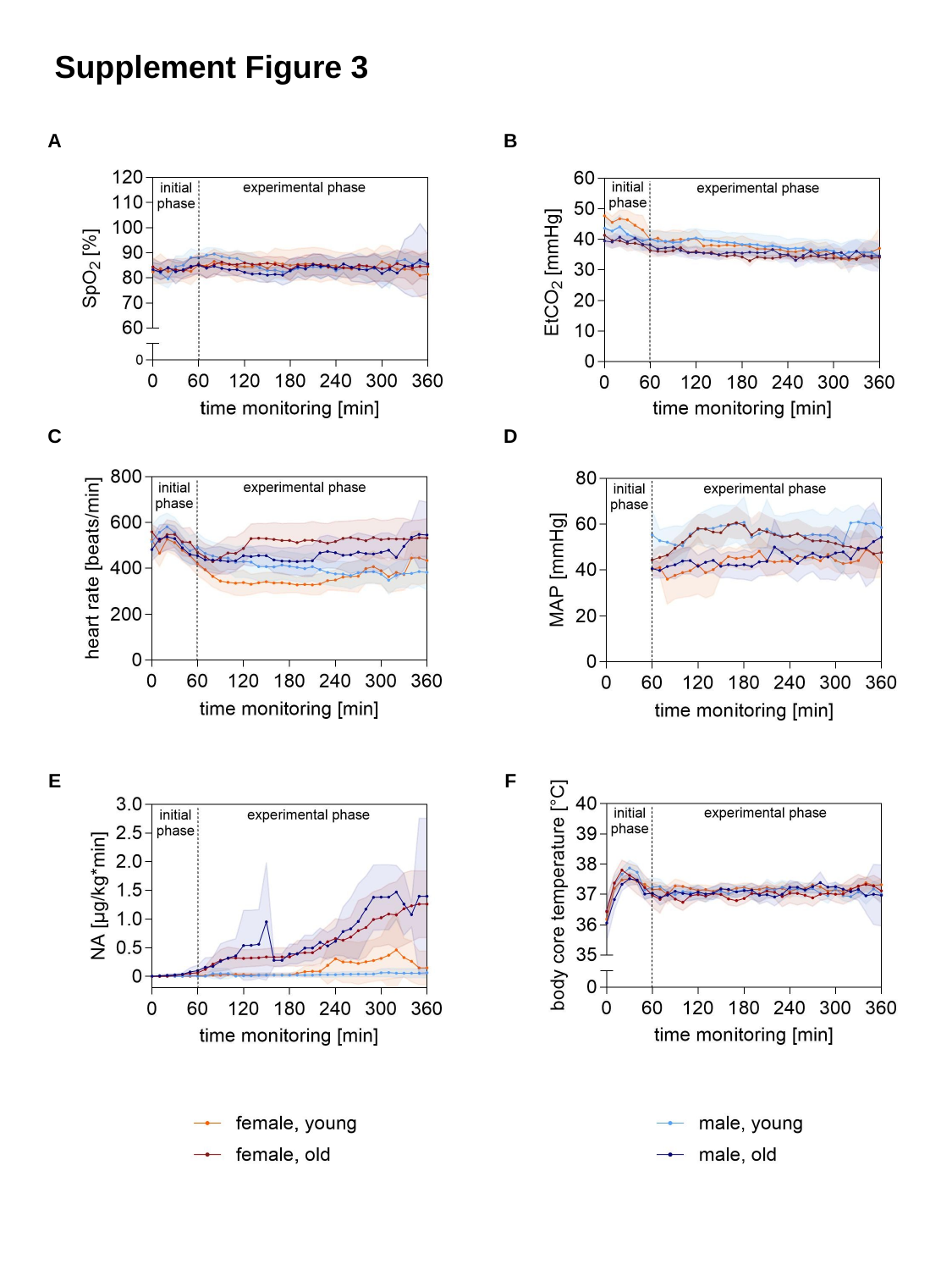

Supplement Figure 3
A
B
C
D
E
F

## Slide 4
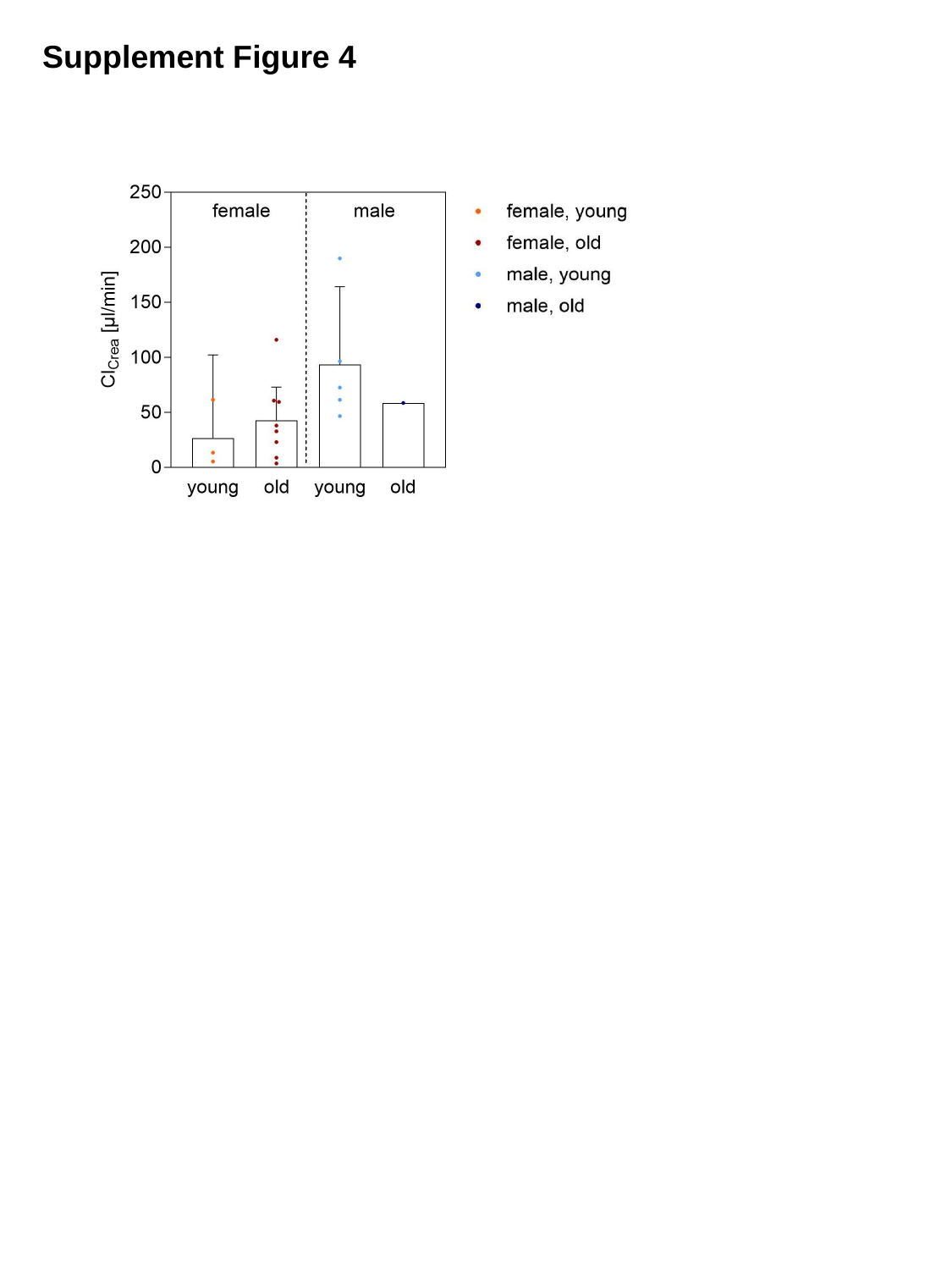

Supplement Figure 4

## Slide 5
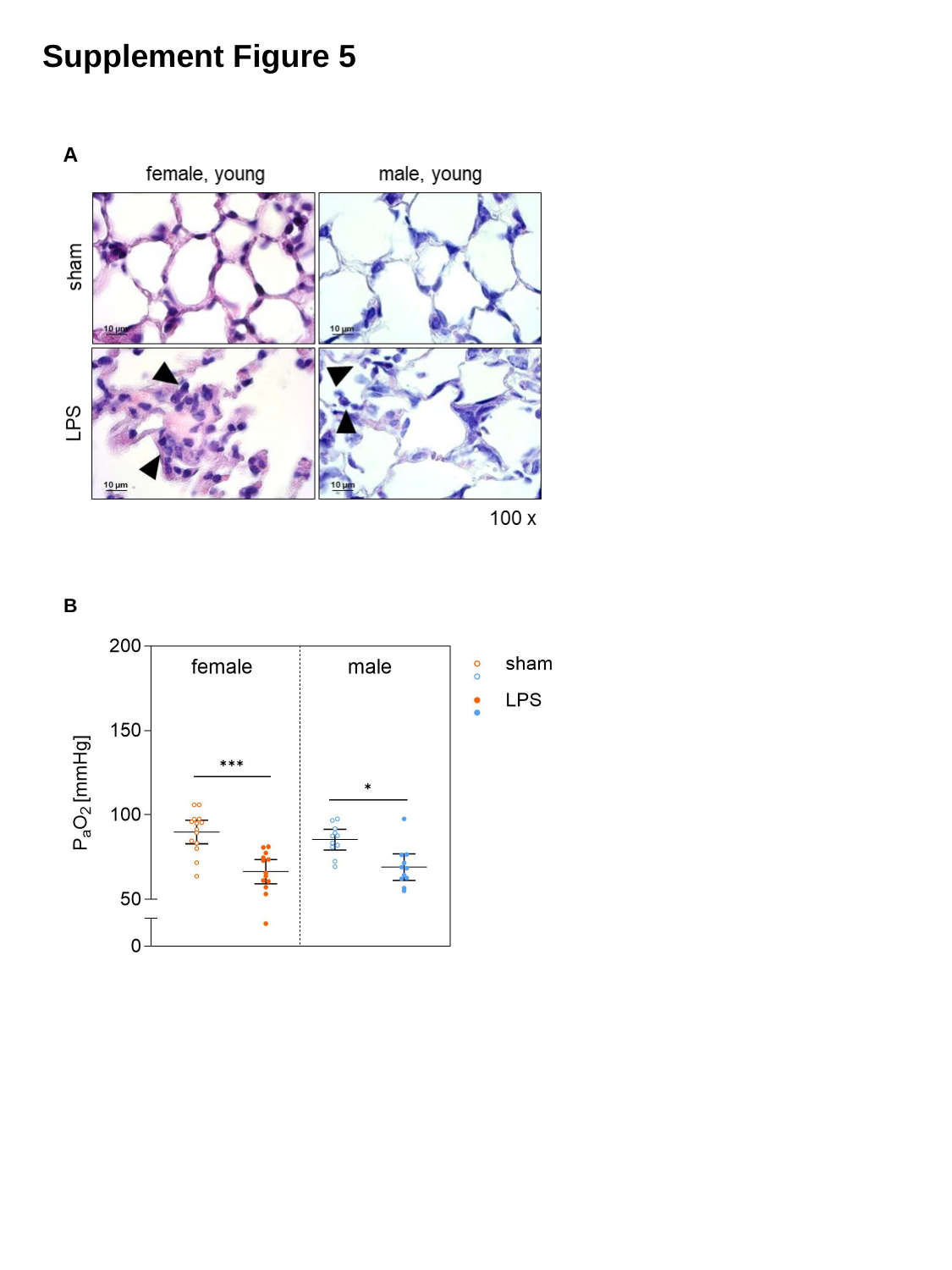

Supplement Figure 5
B
