# Supplementary material for: Sex- and age-related differences in LPS-induced lung injury: establishing a mouse intensive care unit
Source: Intensive Care Med Exp. 2025 May 6;13:48. doi: 10.1186/s40635-025-00756-6 (PMC12055714; doi:10.1186/s40635-025-00756-6)
Supplement: Supplementary file 2 — Supplementary Material 2 [file 40635_2025_756_MOESM2_ESM.docx]

**Supplement**

**Sex- and Age-Related Differences in LPS-Induced Lung Injury: Establishing a Mouse Intensive Care Unit**

Chantal Crispens^1*^, Emilia Fleckenstein^1*^, Annett Wilken-Schmitz^1^, Sandra Weber^2^, Michael Gröger^2^, Andrea Hoffmann^2^, Peter Radermacher^2^, Lucy Kathleen Reiss^3^, Steven R. Talbot^4^, Laura Kästner^5^, Kernt Köhler^5^, Kai Zacharowski^1^, Andreas von Knethen^1^ and Ulrike Heinicke^1^

^1^Goethe University, University Hospital Frankfurt, Department of Anesthesiology, Intensive Care Medicine and Pain Therapy, Theodor-Stern-Kai 7, 60590 Frankfurt am Main, Germany

^2^Institute for Anesthesiological Pathophysiology and Process Engineering, Ulm University, Helmholtzstrasse 8-1, Ulm, Germany

^3^Department of Pharmacology and Toxicology, RWTH Aachen University, 52074 Aachen, Germany

^4^Hannover Medical School, Institute for Laboratory Animal Science, Carl-Neuberg-Straße 1, 30625 Hannover, Germany

^5^Institute of Veterinary Pathology, Justus Liebig University, Frankfurter Str. 96, 35392 Giessen, Germany

^*^These authors share first authorship.

Correspondence:

Dr. Ulrike Heinicke

Goethe University

University Hospital Frankfurt

Department of Anesthesiology, Intensive Care Medicine and Pain Therapy

Theodor-Stern-Kai 7

60590 Frankfurt am Main

Germany

Email: [heinicke@med.uni-frankfurt.de](mailto:heinicke@med.uni-frankfurt.de)

# Supplement Materials and Methods

**Anesthesia, surgical instrumentation, and experimental protocol**

The initiation of each animal into the experiment was carried out according to a fixed procedure (Figure 1). Anesthesia was initially induced intraperitoneally (i.p.) with ketamine, midazolam, and fentanyl (ketamine [120 µg/g body weight (bw)], midazolam [1.25 µg/g bw], and fentanyl [0.25 µg/g bw], 10 ml/kg), followed by placing the mouse in a supine position on a heating mat (Small Animal Physiological Monitoring System (HPMS); Hugo Sachs Electronics, March, Germany) and applying electrocardiogram (ECG) electrodes, a rectal temperature sensor, and a pulse oximeter. The trachea was intubated via tracheotomy (Supplement Figure 1A and B) and lungs were mechanically ventilated with a lung-protective ventilation strategy [1] using a small animal ventilator (MidiVent Ventilator Type 849; Hugo Sachs Electronics, March, Germany), an inspiratory to expiratory (I:E) ratio of 1:1, and a volume-controlled ventilation at initially 8 ml/kg body weight (bw) tidal volume (V_T_), and of 2 cmH_2_O in positive end-expiratory pressure (PEEP) manually set by submerging a glass capillary in water [2, 3]. The V_T_ was increased on demand in response to inadequate oxygen saturation and the presence of ST-segment depressions on the ECG. The respiratory rate (RR) was set at 150–170 breaths per minute. A combination of low V_T_ with 8 ml/kg and respiratory rate of 180/min had been shown to produce adequate minute ventilation and P_a_CO_2_ in mice [3].The inspiratory flow was generated from room air, setting the fraction of inspired oxygen (F_i_O_2_) to 0.21. Recruitment maneuvers (RM) to stabilize lung compliance were employed in a 5-minute interval via lung inflation to a peak inspiratory pressure (P_IP_) of 30 cmH_2_O for one breath [4].

Catheters were inserted into the jugular vein, carotid artery, and bladder (Supplement Figure 1C, D, E, and F), and fluid resuscitation with balanced crystalloids (20 ml/kg bw/h, Sterofundin; B. Braun, Melsungen, Germany) as well as anesthesia were maintained intravenously with ketamine [100 µg/g bw per hour (µg/g*h)], midazolam [0.2 µg/g*h], and fentanyl [1.0 µg/g*h].

Efforts were made to maintain a stable mean arterial pressure (MAP) at approximately ±10 % of the initial baseline value throughout the course, and noradrenaline (NA) titration were administered and escalated on demand [0.03-2 µg/kg*min]. Circulatory failure was defined as a persistent decline in MAP reaching MAP values <30 mmHg without achieving stabilization at any level. Physiological parameters, including heart rate (HR), respiratory parameters, and acid-base status, were monitored over 6 h. The experimentation time was divided into an “initial phase” (t = 60 min), containing the completion of all surgical interventions, and the remaining time as “experimental phase” (t = 60 ≤ 360 min). Body core temperature was measured rectally, peripheral oxygen saturation (SpO_2_) photometrically, cardiac action via electrocardiograph (Small Animal Physiological Monitoring System (HPMS); Hugo Sachs Electronics, March, Germany), and HR by calculation via ECG (Supplement Figure 1G). To evaluate MV during the experiment, we measured end-tidal carbon dioxide (EtCO_2_) via capnography (Capnograph for small rodents type 340; Hugo Sachs Electronics, March, Germany), PEEP and P_IP_ (via LabChart Software). Mean values of V_t_ from the MICU establishment were applied for the 1-hour MICU.

Urine was collected via the catheter and creatinine levels were determined (Crea) via enzyme-linked immunosorbent assay (ELISA) (Mouse Creatinine Assay Kit No. MBS763433; MyBioSource, San Diego, USA) in urine and plasma. Then, creatinine clearance was calculated via (Crea_urine_ [mg/dL] * total urine volume [mL]) / (Crea_plasma_ [mg/dL] * t[min]) (Supplement Figure 3). For the LPS model, anesthesia was administered on demand via i.p. injection, with monitoring limited to one hour and no catheter placement.

Finally, mice were sacrificed by a cut of the *carotid artery*, followed by cervical dislocation. The cardiovascular system was flushed with phosphate buffered saline (PBS) for 3 minutes and the left lobe of the lung was perfused with 4 % formaldehyde (PFA) and then fixed for 2 h at room temperature (RT) for histological analyses. All other organs were removed and frozen at -80 °C.

# Supplement results

For MICU establishment, 56 mice entered the phase of MV and were included in the analysis. A survival curve of all groups demonstrated that due to technical and surgical issues, four mice were eliminated from the experiment in the initial phase (male young n = 1, female young n = 2, female old n = 1). Furthermore, 25 mice dropped out at a later stage in the experimental phase due to cardiocirculatory failure (Supplement Figure 1H). Notably, these losses were predominantly seen in old males (male, young n = 4, survival = 64.3 %; female, young n = 6, survival = 42.9 %; male, old n = 11, survival = 21.4 %; female, old n = 4, survival = 64.3 %).

MV, RR and V_T_ were set manually and resulted in stable P_IP_ levels in all groups in the experimental phase (Supplement Figure 2A-C). Mice needed higher V_T_ in the experimental than in the initial phase (p < 0.001) (Supplement Figure 2B). V_T_ was higher in females than males (p = 0.0201) and in older than younger (p = 0.0317) mice. PEEP levels were stable throughout the experimental phase in all groups (Supplement Figure 2D). RR did not differ between sex, age, or time points (Supplement Figure 2C). SpO_2_ was improved in the experimental phase compared to the initial phase (initial phase 84 (83-84) %, experimental phase 85 (84-85) %; p = 0.007) and then held stable (Supplement Figure 3A). EtCO_2_ differed between phases dependent on both sex and age (p = 0.001). After connection to the ventilator, initially elevated EtCO_2_ approached normal values (Supplement Figure 3B). It further decreased subtly in the experimental phase (p < 0.001) but stayed in physiological range of 30-45 mmHg in all groups. Static pulmonal compliance was constant in all groups (Supplement Figure 2E).

All mice showed variations in HR in the initial phase, which disappeared when anesthesia was supplied i.v. rather than i.p. (Supplement Figure 3C). HR differed between phases in both young (p < 0.001) and old mice (p < 0.001). Old mice tended to show higher HR compared to young mice (male young 424 (405-444), male old 468 (456-480), female young 385 (365-405), and female old 514 (504-524) beats/min). MAP was dependent on the measurement time (p < 0.001) and highest in old females and young males (Supplement Figure 3D). MAP was lower in old males and young females compared to old females (p = 0.001, p < 0.001) and young males (p < 0.001, p < 0.001) (male young 56 (55-58), male old 45 (44-46), female young 43 (42-45), and female old 53 (53-55) mmHg). Concerning cardiocirculatory support, old mice needed more and increasing (p < 0.001) NA therapy than young mice (male young 0.03 (0.02-0.04), male old 0.61 (0.45-0.77), female young 0.12 (0.08-0.17), and female old 0.51 (0.38-0.64) [µg/kg*min]) (Supplement Figure 3E). Temperature was held stable over time and across all groups (Supplement Figure 3F).

BGAs were obtained from all mice at the beginning and the end of the 6-hour MICU (Supplement Table 1, 2). All animals exhibited an acidotic pH at the start of the experiment, with acidosis intensifying, particularly in young females. Acidosis correlated with lower BE, increased potassium, and reduced bicarbonate across all groups, though potassium increases were most pronounced in young females. Lactate levels started elevated but tended toward normal, while P_a_CO₂, P_a_O₂, and S_a_O₂ levels remained stable. Chloride levels rose over time in most groups, except young males, and glucose levels declined in all groups by the end of the 6-hour MICU (Supplement Table 1, 2). Furthermore, creatinine clearance did not differ between groups (Supplemental Figure 4).

In summary, the results of the MICU establishment revealed sex- and age-related differences in physiological responses during MV, particularly in HR, blood pressure, and the need for cardiocirculatory support, with old males showing the highest mortality and requiring more NA to maintain stable hemodynamics. Despite initial variations, respiratory parameters such as SpO_2_ and P_IP_ were stabilized across all groups during the experimental phase. However, females required higher V_T_ than males, and old mice needed higher V_T_ than young mice.

BGA results indicated that the high infusion volumes relative to the mice’s bw caused blood acidification due to the elevated Cl^-^ concentration (127 mmol/L) in the crystalloid solution. The Cl^-^ concentration of the infusion solution aligned with the measured values, which showed an approximate increase of 10 mmol/L, reducing the anion gap accordingly (Supplement Table 1). The modest change in BE likely reflected probably partial compensation through hemodilution (Hb decrease of ~2 g/dL) and albumin dilution. These findings suggested that either significantly lower infusion volumes should be used and/or a crystalloid solution different in electrolyte distribution should be infused in future experiments to minimize adverse effects.

# Supplement Tables

Supplement Table 1 Initial and final BGA results of the MICU establishment

| **parameter** | **female, young** | | **female, old** | | **male, young** | | **male, old** | |
| --- | --- | --- | --- | --- | --- | --- | --- | --- |
|  | **initial** | **final** | **initial** | **final** | **initial** | **final** | **initial** | **final** |
| pH [-log_10_c(H^+^)] | 7.17 (7.12-7.23) | 7.08 (7.01-7.15) | 7.27 (7.21-7.33) | 7.12 (7.05-7.18) | 7.3 (7.22-7.37) | 7.21 (7.16-7.25) | 7.21 (7.15-7.26) | 7.12 (7.03-7.20) |
| P_a_O_2_ [mmHg] | 92 (69-116) | 80 (62-97) | 84 (71-97) | 97 (86-108) | 94 (74-113) | 86 (75-97) | 82 (72-90) | 93 (62-125) |
| P_a_CO_2_ [mmHg] | 55 (47-63) | 51 (39-64) | 42 (32-52) | 34 (29-39) | 37 (30-44) | 39 (34-45) | 46 (40-52) | 37 (26-49) |
| S_a_O_2_ [%] | 71 (54-88) | 78 (38-118) | 91 (79-92) | 87 (82-92) | 89 (83-94) | 86 (79-92) | 84 (79-90) | 86 (29-143) |
| Hb [g/dl] | 12.6 (10.4-14.8) | 9,0 (6.7-11.3) | 12.4 (11.5-13.2) | 9.7 (9.2-10.3) | 11.4 (9.6-13.3) | 9.1 (8.4-9.8) | 10.3 (9.8-10.8) | 9.3 (4.7-14.0) |
| K^+^ [mmol/L] | 5.4 (5.2-5.6) | 8.4 (7.6-9.2) | 4.9 (4.5-5.3) | 6.3 (5.3-7.2) | 5.4 (4.7-6.0) | 5.9 (5.3-6.4) | 4.5 (4.3-4.8) | 6.8 (6.1-7.5) |
| Na^+^ [mmol/L] | 150 (145-155) | 150 (149-151) | 148 (146-151) | 151 (150-153) | 153 (146-160) | 158 (155-161) | 152 (151-154) | 153 (149-157) |
| Ca^2+^ [mmol/L] | 1.4 (1.2-1.5) | 1.2 (1.2-1.3) | 1.4 (1.3-1.4) | 1.2 (1.2-1.5) | 1.4 (1.2-1.5) | 1.3 (1.2-1.3) | 1.3 (1.3-1.4) | 1.2 (1.0-1.4) |
| Cl^-^ [mmol/L] | 121 (116-127) | 130 (127-134) | 122 (119-124) | 134 (133-135) | 126 (118-134) | 136 (134-138) | 124 (121-126) | 135 (131-139) |
| Glu [mmol/L] | 9.4 (8.2-10.7) | 5.7 (3.4-8) | 10.7 (9.5-11.9) | 5.2 (4.2-6.2) | 9.2 (7.1-11.2) | 5 (4.3-5.6) | 9.2 (7.8-10.7) | 5.6 (-1.6-12.6) |
| Lac [mmol/L] | 1.2 (0.9-1.5) | 1.0 (0.7-1.3) | 1.3 (1.0-1.6) | 1.5 (1.1-2.0) | 1.5 (0.8-2.1) | 0.7 (0.5-0.8) | 2.5 (1.8-3.1) | 1.7 (0.9-2.4) |
| BE [mmol/L] | -8.3 (-10.9-  -5.7) | -14.8 (-16.1-  -13.4) | -8.5 (-10.9-  -6.0) | -18.4 (-21.1-  -15.7) | -8.5 (-12.1-  -5.0) | -12.4 (-14.0-  -11.2) | -9.7 (-11.9-  -7.5) | -17.4 (-18.4-  -16.4) |

Data are shown as means ± 95 % CI. BGA, blood gas analysis; pH, potential of hydrogen; P_a_O_2_, partial pressure of arterial oxygen; P_a_CO_2_, partial pressure of arterial carbon dioxide; S_a_O_2_, oxygen saturation; Hb, haemoglobin; K^+^, potassium; Na^+^, sodium; Ca^2+^, calcium; Cl^-^, chloride; Glu, glucose; Lac, lactate; HCO_3_^-^, bicarbonate; BE, base excess.

Supplement Table 2 p-values of the initial and final BGA results of the MICU establishment

| **parameter** | **f, young *vs* f, old** | | **m, young *vs* m, old** | | **m, young *vs* f, young** | | **m, old *vs* f, old** | |
| --- | --- | --- | --- | --- | --- | --- | --- | --- |
|  | **initial** | **final** | **initial** | **final** | **initial** | **final** | **initial** | **final** |
| pH | ns | ns | ns | ns | 0.018 | 0.036 | ns | ns |
| P_a_O_2_ | ns | ns | ns | ns | ns | ns | ns | ns |
| P_a_CO_2_ | ns | 0.011 | ns | ns | 0.046 | ns | ns | ns |
| S_a_O_2_ | 0.002 | ns | ns | ns | 0.006 | ns | ns | ns |
| Hb | ns | ns | ns | ns | ns | ns | ns | ns |
| K^+^ | ns | <0.001 | ns | ns | ns | <0.001 | ns | ns |
| Na^+^ | ns | ns | ns | ns | ns | ns | 0.038 | ns |
| Ca^2+^ | ns | ns | ns | ns | ns | ns | ns | ns |
| Cl^-^ | ns | ns | ns | ns | ns | ns | ns | ns |
| Glu | ns | ns | ns | ns | ns | ns | ns | ns |
| Lac | ns | ns | 0.006 | ns | ns | ns | <0.001 | ns |
| BE | ns | ns | ns | ns | ns | ns | ns | ns |

P-values are calculated with One-way ANOVA for normality-distributed data and Kruskal-Wallis test for non-normality distributed data using GraphPad Prism 10.0.2; *p ˂ 0.05, **p ˂ 0.01, ***p ˂ 0.001. BGA, blood gas analysis; *vs*, versus; pH, potential of hydrogen; P_a_O_2_, partial pressure of arterial oxygen; P_a_CO_2_, partial pressure of arterial carbon dioxide; S_a_O_2_, oxygen saturation; Hb, haemoglobin; K^+^, potassium; Na^+^, sodium; Ca^2+^, calcium; Cl^-^, chloride; Glu, glucose; Lac, lactate; HCO_3_^-^, bicarbonate; BE, base excess.

Supplement Table 3 BGA results after LPS stimulation and MV on the 1-hour MICU

| **parameters** | **female, young** | | **female, old** | | **male, young** | | **male, old** | |
| --- | --- | --- | --- | --- | --- | --- | --- | --- |
|  | **sham** | **LPS** | **sham** | **LPS** | **sham** | **LPS** | **sham** | **LPS** |
| pH [-log_10_c(H^+^)] | 7.27 (7.22-7.31) | 7.22 (7.17-7.27) | 7.31 (7.28-7.34) | 7.18 (7.02-7.35) | 7.30 (7.25-7.36) | 7.29 (7.23-7.34) | 7.35 (7.32-7.38) | 7.19 (7.08-7.29) |
| P_a_O_2_ [mmHg] | 93 (85-102) | 86 (78-94) | 90 (87-94) | 102 (75-129) | 93 (88-99) | 90 (79-101) | 93 (85-101) | 108 (101-114) |
| P_a_CO_2_ [mmHg] | 43 (37-50) | 50 (42-57) | 42 (40-45) | 41 (36-47) | 40 (35-44) | 47 (43-51) | 38 (35-41) | 34 (28-41) |
| S_a_O_2_ [%] | 89 (85-93) | 85 (80-90) | 91 (89-92) | 90 (83-97) | 90 (87-92) | 86 (81-91) | 91 (88-94) | 91 (89-93) |
| Hb [g/dl] | 13.6 (13.4-13.9) | 14.6 (14.1-15.1) | 13.4 (12.9-13.8) | 14.6 (12.6-16.5) | 13.4 (12.6-14.1) | 14.1 (13.5-14.8) | 13.3 (12.8-13.8) | 14.4 (13.6-15.2) |
| K^+^ [mmol/L] | 5.7 (5.4-6) | 5.4  (5-5.8) | 5.1 (4.8-5.4) | 5.5 (4.5-6.5) | 7.4 (2.9-11.9) | 4.9 (4.6-5.1) | 5.3  (5-5.5) | 9  (1.9-16.1) |
| Na^+^ [mmol/L] | 147 (146-147) | 146 (146-147) | 148 (147-148) | 146 (144-148) | 148 (145-150) | 148 (146-150) | 149 (148-150) | 145 (142-149) |
| Ca^2+^ [mmol/L] | 1.3 (1.3-1.3) | 1.2 (1.2-1.2) | 1.2 (1.2-1.3) | 1.2 (1.2-1.3) | 1.2 (1.2-1.3) | 1.2 (1.2-1.3) | 1.3 (1.3-1.3) | 1.3 (1.3-1.4) |
| Cl^-^ [mmol/L] | 119 (118-119) | 116 (114-117) | 118 (116-119) | 118 (113-123) | 119 (117-121) | 117 (111-123) | 118 (116-119) | 120 (118-122) |
| Glu [mmol/L] | 9.4 (8.5-10.3) | 7.3 (5.8-8.8) | 10.1 (9.5-10.7) | 11.2 (7.2-15.1) | 9.3 (7.9-10.8) | 8.1 (6.5-9.7) | 10.1 (9.4-10.9) | 11.1 (8-14.1) |
| Lac [mmol/L] | 0.5 (0.4-0.6) | 1  (0.7-1.3) | 0.7 (0.5-0.8) | 2.3  (-0.4-5.1) | 0.6 (0.5-0.6) | 0.7 (0.6-0.9) | 0.8 (0.6-0.9) | 3.3 (1.3-5.2) |
| BE [mmol/L] | -7.80  (-8.6-  -7.1) | -7.70  (-9.2-  -6.3) | -5.0  (-6.2-  -3.9) | -12.2  (-20.9-  -3.5) | -6.8  (-8.3-  -5.2) | -4.3  (-6.1-  -2.4) | -4.5  (-6.6-  -2.5) | -14.4 (-20.8-  -8.1) |

Data are shown as means ± 95 % CI. BGA, blood gas analysis; pH, potential of hydrogen; P_a_O_2_, partial pressure of arterial oxygen; P_a_CO_2_, partial pressure of arterial carbon dioxide; S_a_O_2_, oxygen saturation; Hb, haemoglobin; K^+^, potassium; Na^+^, sodium; Ca^2+^, calcium; Cl^-^, chloride; Glu, glucose; Lac, lactate; BE, base excess.

Supplement Table 4 p-values of the BGA results after LPS stimulation and MV on the 1-hour MICU

| **parameters** | **young** | | | **old** | | | **young *vs* old** | |
| --- | --- | --- | --- | --- | --- | --- | --- | --- |
|  | **female LPS *vs* male LPS** | **female LPS *vs* female sham** | **male LPS *vs* male sham** | **female LPS *vs* LPS male** | **female LPS *vs* female sham** | **male LPS *vs* male sham** | **females LPS: young *vs* old** | **males LPS: young *vs* old** |
| pH | ns | ns | ns | ns | ns | 0.006 | ns | ns |
| P_a_O_2_ | ns | ns | ns | ns | ns | ns | ns | 0.014 |
| P_a_CO_2_ | ns | ns | ns | ns | ns | ns | ns | 0.015 |
| S_a_O_2_ | ns | ns | ns | ns | ns | ns | ns | ns |
| Hb | ns | ns | ns | ns | ns | ns | ns | ns |
| K^+^ | ns | ns | ns | ns | ns | ns | ns | 0.028 |
| Na^+^ | ns | ns | ns | ns | ns | ns | ns | ns |
| Ca^2+^ | ns | 0.027 | ns | ns | ns | ns | ns | ns |
| Cl^-^ | ns | ns | ns | ns | ns | ns | ns | ns |
| Glu | ns | ns | ns | ns | ns | ns | ns | ns |
| Lac | ns | 0.025 | ns | ns | ns | ns | ns | ns |
| BE | ns | ns | ns | ns | ns | 0.007 | ns | 0.003 |

P-values are calculated with the Kruskal-Wallis test using GraphPad Prism 10.0.2; *p ˂ 0.05, **p ˂ 0.01, ***p ˂ 0.001. BGA, blood gas analysis; *vs*, versus; pH, potential of hydrogen; P_a_O_2_, partial pressure of arterial oxygen; P_a_CO_2_, partial pressure of arterial carbon dioxide; S_a_O_2_, oxygen saturation; Hb, haemoglobin; K^+^, potassium; Na^+^, sodium; Ca^2+^, calcium; Cl^-^, chloride; Glu, glucose; Lac, lactate; BE, base excess.

Supplement Table 5 Score Sheet of intratracheal LPS application

**date/time and volume of intratracheal application:**

| **mouse number:** | □ LPS □ Sham | | |  |  |  |  |
| --- | --- | --- | --- | --- | --- | --- | --- |
| **hours after i.t. application** | **2** | **4** | **6** | **12** | **24** |  |  |
| **behavior** |  |  |  |  |  | 0 | inconspicuous, active, interested |
|  |  |  |  |  |  | 0.2 | calm, runs away after being touched |
|  |  |  |  |  |  | 0.4 | apathetic, does not react after being touched |
| **weight** |  |  |  |  |  | 0 | stable or increased |
|  |  |  |  |  |  | 0.2 | weight loss 5-10 % |
|  |  |  |  |  |  | 0.4 | weight loss > 10 % |
| **visual appearance** |  |  |  |  |  | 0 | smooth, shiny coat |
|  |  |  |  |  |  | 0.2 | slightly ruffled coat |
|  |  |  |  |  |  | 0.4 | dull, ruffled coat |
| **mobility** |  |  |  |  |  | 0 | moves normally |
|  |  |  |  |  |  | 0.2 | movement reduced, pain evident when walking |
|  |  |  |  |  |  | 0.4 | lies cowering in the corner |
| **breathing** |  |  |  |  |  | 0 | normal |
|  |  |  |  |  |  | 0.2 | fast, shallow |
|  |  |  |  |  |  | 0.6 | forced, heavy |
| **total score** |  |  |  |  |  |  |  |

**Cancellation criterion:**

**Score ≥ 1**: Mouse is removed from the experiment and euthanized adequately without pain or immediately admitted to the MICU for one-hour ventilation and diagnostics.

Supplement References

1. Uhlig U, Uhlig S (2011) Ventilation-induced lung injury. Compr Physiol 1:635–661. https://doi.org/10.1002/cphy.c100004

2. Hemmes SNT, Serpa Neto A, Schultz MJ (2013) Intraoperative ventilatory strategies to prevent postoperative pulmonary complications: a meta-analysis. Curr Opin Anaesthesiol 26:126–133. https://doi.org/10.1097/ACO.0b013e32835e1242

3. Mekontso Dessap A, Voiriot G, Zhou T et al. (2012) Conflicting physiological and genomic cardiopulmonary effects of recruitment maneuvers in murine acute lung injury. Am J Respir Cell Mol Biol 46:541–550. https://doi.org/10.1165/rcmb.2011-0306OC

4. Reiss LK, Kowallik A, Uhlig S (2011) Recurrent recruitment manoeuvres improve lung mechanics and minimize lung injury during mechanical ventilation of healthy mice. PLoS One 6:e24527. https://doi.org/10.1371/journal.pone.0024527
